# Do settlement dynamics influence competitive interactions between an alien tunicate and its native congener?

**DOI:** 10.1002/ece3.2655

**Published:** 2016-12-17

**Authors:** Sarah Bouchemousse, Laurent Lévêque, Frédérique Viard

**Affiliations:** ^1^ Sorbonne UniversitésUPMC Univ Paris 6Station Biologique de Roscoff29680 RoscoffFrance; ^2^ CNRSUMR7144Adaptation et Diversité en Milieu MarinEquipe DIVCOStation Biologique de Roscoff29680 RoscoffFrance; ^3^ CNRS FR2424Station Biologique de Roscoff29680 RoscoffFrance

**Keywords:** *Ciona* spp., congeneric species, long‐term coexistence, marine invaders, settlement

## Abstract

Variation in density of early stages, that is, larvae and juveniles, is a major determinant of the distribution and abundance of the adult population of most marine invertebrates. These early stages thus play a key role in competitive interactions, and, more specifically, in invasion dynamics when biologically similar native and non‐native species (NNS) come into contact in the same habitat. We examined the settlement dynamics and settlement rate of two important members of the fouling community that are common on human‐made infrastructures around the world: *Ciona robusta* (formerly known as *Ciona intestinalis* type A) and *C. intestinalis* (formerly known as *C. intestinalis* type B). In the western English Channel, the two species live in close syntopy following the recent introduction of *C. robusta* in the native European range of *C. intestinalis*. Using settlement panels replaced monthly over 2 years in four marinas (including one studied over 4 years) and species‐diagnostic molecular markers to distinguish between juveniles of both species (*N* = 1,650), we documented similar settlement dynamics of both species, with two settlement periods within a calendar year. With one exception, settlement times were highly similar in the congeners. Although the NNS showed lower settlement density than that of the native congener, its juvenile recruitment was high during the second settlement period that occurs after the warm season, a pattern also observed in adult populations. Altogether, our results suggest that species’ settlement dynamics do not lead to the dominance of one species over the other through space monopolization. In addition, we showed that changes over time are more pronounced in the NNS than in the native species. This is possibly due to a higher sensitivity of the NNS to changes of environmental factors such as temperature and salinity. Environmental changes may thus eventually modify the strength of competitive interactions between the two species as well as species dominance.

## 1 Introduction

Coastal man‐made infrastructures, such as harbors, marinas, or aquaculture facilities, are growing in number and in size and are modifying marine coastal landscapes at an unprecedented rate around the world (Airoldi & Beck, 2007; Bulleri & Chapman, 2010). Ballast waters and hull fouling are major introduction vectors of non‐native species (NNS) in marine systems, making coastal man‐made infrastructures the main gateways and bridgeheads for biological invasions in marine ecosystems (Airoldi, Turon, Perkol‐Finkel, & Rius, 2015; Briggs, 2012; Lopez‐Legentil, Legentil, Erwin, & Turon, 2015). Many NNS have become established in these artificial habitats (Lambert & Lambert, 1998; Ruiz, Freestone, Fofonoff, & Simkanin, 2009; Tyrrell & Byers, 2007). In the English Channel, NNS may represent up to 25% of the sessile fauna in marinas (Arenas et al., 2006), with new arrivals recorded as recently as last year (Bishop, Wood, Leveque, Yunnie, & Viard, 2015).

One important consequence of biological introductions is that they disrupt the natural (spatial) barriers that prevent competitive interactions between closely related taxa, including congeners. Anthropogenic secondary contacts between congeners have been described for several important members of the fouling community observed on human‐made infrastructures. Tunicates are one notable example with the coexistence of the NNS *Botrylloides violaceus* with its native congener *Botrylloides diegensis* or three non‐native congeners of *Styela* (i.e., *Styela clava*,* Styela plicata,* and *Styela canopus*) along the western coast of North America (Lambert & Lambert, 1998, 2003). When congeners display similar biological traits and ecological niches, competitive processes are expected to be intensified (e.g., in the marine environment, between *Idotea* species, Franke, Gutow, & Janke, 2007, for a review, see Branch, 1984). Competitive interactions can rapidly modify the distribution of both native and NNS in the newly colonized environment (Fridley et al., 2007; Stachowicz, Whitlatch, & Osman, 1999). Strong competitive interactions between congeners, leading to species exclusion, have been demonstrated in marine systems, for example, in the *Mytilus* species complex in California for which there has been a range shift of the native mollusk species *M. trossolus*, directly attributable to the introduction of *M. galloprovincialis* (Lockwood & Somero, 2011).

Many marine NNS are invertebrates (Molnar, Gamboa, Revenga, & Spalding, 2008), most of which have a bentho‐pelagic life cycle (i.e., a dispersing pelagic larval stage alternating with an adult sessile stage). As emphasized by the supply‐side ecology (Gaines & Roughgarden, 1985; Lewin, 1986), the supply of larvae and the recruitment success of postsettlement stages (juveniles) are key factors influencing adult abundance and distribution (e.g., Edwards & Stachowicz, 2011, 2012; Hunt & Scheibling, 1997; Pechenik, Li, & Cochrane, 2002; Roughgarden, Gaines, & Possingham, 1988). As major components of the propagule pressure, these larval and juvenile stages are also critical for the invasion success or failure (Simberloff, 2009). For example, larval supply of the invasive slipper limpet *Crepidula fornicata* may limit its local proliferation (Rigal, Viard, Ayata, & Comtet, 2010). The timing of settlement may also facilitate a shift in dominance, favoring NNS at the community scale (Stachowicz, Terwin, Whitlatch, & Osman, 2002). The presence of juveniles can in addition prevent settlement or increase postsettlement mortality in closely related species (e.g., between native and non‐native tunicates; Rius, Turon, & Marshall, 2009). Variation in settlement rate can thus be critical in determining the type and intensity of the interactions between biologically similar congeners living in the same locations and habitats (i.e., syntopy).

We here compared the settlement dynamics of two emblematic tunicate species, for which the taxonomic status has been recently re‐evaluated (Brunetti et al., 2015): *Ciona robusta* and *Ciona intestinalis* (formerly named *C. intestinalis* type A and *C. intestinalis* type B, respectively). The two species are important members of fouling communities and well established in artificial habitats including marinas and harbors worldwide (Aldred & Clare, 2014; Bouchemousse, Lévêque, Dubois, & Viard, 2016; Lambert & Lambert, 1998; Ramsay, Davidson, Landry, & Arsenault, 2008; Tracy & Reyns, 2014). They live in syntopy in several marinas in the western English Channel and southern Brittany in the Northeast Atlantic (Bouchemousse, Lévêque, et al., 2016; Nydam & Harrison, 2011). This is, so far, the only confirmed region where both species coexist (Bouchemousse, Bishop, & Viard, 2016; Caputi et al., 2007; Zhan, Macisaac, & Cristescu, 2010). Their coexistence in this region is due to the recent introduction of *C. robusta* in the native European range of *C. intestinalis* (ca. 15–20 years ago; Bishop, Wood, Yunnie, & Griffiths, 2015; Nydam & Harrison, 2011). In this region, *C. robusta* has never been reported outside marinas (L.L., personal observation). In the English Channel, *C. intestinalis* is occasionally found in natural habitats (L.L. and F.V., personal observation) although its occurrence and abundance in natural habitats is very low as compared with marinas where flourishing populations develop—and often become dominant in the fouling communities.

Interestingly, the two species are interfertile in laboratory conditions (Bouchemousse, Lévêque, et al., 2016; Suzuki, Nishikawa, & Bird, 2005), but only a few F1 hybrids have been observed in the wild and there is so far no convincing evidence of contemporary introgressions between the two species (Bouchemousse, Haag‐Liautard, Bierne, & Viard, 2016; Bouchemousse, Lévêque, et al., 2016; Nydam & Harrison, 2011). These results suggest the existence of efficient reproductive isolation mechanisms in the wild. Competitive interactions may thus be more important than evolutionary interactions (e.g., adaptive introgression) in determining the fate of these two congeneric species in their sympatric range. However, due to their recent taxonomic revision (Brunetti et al., 2015), their specific life cycle, environmental preferences, and population dynamics have yet to be examined in detail.

In a previous study (Bouchemousse, Lévêque, et al., 2016), we documented substantial seasonal variation in the relative abundance of adult populations of the NNS *C. robusta* compared with its congener, in the English Channel and Atlantic coasts of Brittany. In particular, we showed a very low relative abundance of *C. robusta* in spring compared with autumn. This variation can be attributed to differential settlement success and/or postsettlement competition according to temperature conditions, favoring *C. robusta* during the warmer season and the native species *C. intestinalis* during the colder season. However, data are scarce on the life cycle and settlement dynamics of the two *Ciona* species in Brittany. *Ciona* species usually display a short life cycle, as documented for the two targeted species in other regions (e.g., one or two generations per year for *C. intestinalis* in Sweden (Dybern, 1965), and at least two generations per year for *C. robusta* in the Mediterranean Sea; Caputi, Crocetta, Toscano, Sordino, & Cirino, 2015). Based on the literature, we hypothesized that one to two settlement events per year occur in Brittany. Thus, considering putative environmental preferences and the observed variation in adult abundances of the two species (Bouchemousse, Lévêque, et al., 2016), the timing and intensity of settlement may differ between the two species during a year. The existence of a phenological shift may ultimately lead to an increase in competition between the two species, giving the advantage to the species settling first.

To test for differential postsettlement patterns, we gathered data monthly on the settlement dynamics of the two *Ciona* species in four marinas over 2 years (over 4 years in one of them) on settlement panels. Species‐diagnostic molecular markers were used to reliably identify species at the juvenile stage.

## 2 Materials and Methods

### 2.1 Experimental design and *Ciona* spp. counts

**Figure 1:**
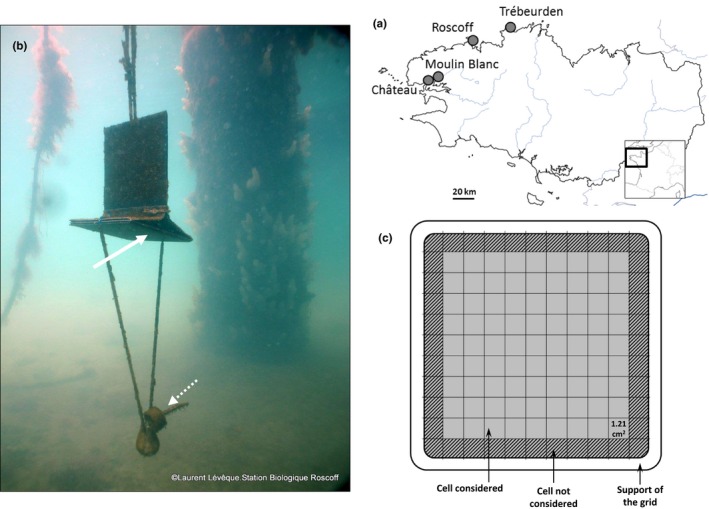
(a) Location of the study sites. Map was downloaded from http://d-maps.com/carte.php?num_car=2,765&lang=fr. (b) Settlement panels employed for the experiment. Juveniles of *Ciona* spp. were counted and sampled on the horizontal panel (indicated by the white arrow). The dotted arrow indicates a temperature data logger (TidbiT^®^v2). Photograph credit: Laurent Lévêque. (c) Counts were carried out on a grid of 11 × 11 cells. Hatched cells along the border of the panel were not considered to avoid edge effects

The settlement dynamics of *Ciona* spp. were investigated in four marinas located along the northern coasts of Brittany and the Bay of Brest (Figure 1a). Previous observations in 2012 (see Table S1 for details) showed that the four marinas harbor various proportions of adult‐stage *C. robusta* relative to *C. intestinalis*: The Roscoff and Château marinas are characterized by low to moderate relative proportions of *C. robusta,* and Trébeurden and Moulin Blanc marinas are characterized by moderate to high relative proportions of *C. robusta* (Bouchemousse, Lévêque, et al., 2016; Table S1 for details).

In each marina, three panels (Figure 1b), located at 2‐m intervals from each other, were placed at 1.5 m depth under a pontoon, close to the local *Ciona* spp. adult population. Each panel had a horizontal surface (15 × 15 cm, Figure 1b) to provide a substrate orientated in the same direction as the pontoons. They were deployed without preconditioning, that is, not soaked in sea water, to allow natural biofilm to develop. They were replaced every month; therefore, settled juveniles were at most 4 weeks old. Note that with such monthly replacement, we did not examine properly the very early postsettlement stages (e.g., a few days old) during which several processes can occur (e.g., predation, competition). The panels were secured and transported back to the laboratory in a cooler filled with seawater taken from the marina. In all marinas, panels were retrieved and brought back to the laboratory for treatment each month between March 2013 and March 2015 (i.e., a 25‐month dataset). In Moulin Blanc, the experimental design started much earlier, with the first panel series set up in November 2010 (and retrieved in December 2010) and the last one retrieved in March 2015. In this marina, the settlement dynamics were thus investigated over 52 months labeled from 0 (December 2010) to 51 (March 2015). In the laboratory, for each panel, the number of juveniles was recorded under a stereomicroscope using a grid composed of 11 × 11 cells of 1.21 cm^2^ each (1.1 × 1.1 cm, Figure 1c). To avoid edge effects, the first row of cells along the edges was excluded; thus, only 81 cells were used for juvenile counts (Figure 1c). Panels were counted on the day or the day after their collection in the field.

To compare the density of the two species between juvenile and adult stages, samples of adult *Ciona* spp. populations were carried out in all four marinas in spring and autumn 2014 (i.e., May and October). These adult populations likely correspond to two different generations and to parents of the juveniles that settled during the year. Sampling was carried out by scuba‐diving under two adjacent pontoons, close to the settlement panels using 10 quadrats of 0.1 m^2^ (40 × 25 cm) per pontoon along a 50‐m‐long transect with one quadrat every 5 m. Individuals sampled were identified at the species level using the morphological criteria described in Sato, Satoh, and Bishop (2012).

### 2.2 Sampling and molecular species identification of the juveniles

Due to their small size and large numbers, the juveniles of *C. robusta* and *C. intestinalis* cannot be reliably discriminated morphologically. A species‐diagnostic marker was thus used on a subsample of juveniles collected on the panels in 2013 and 2014 to estimate the proportion of the two species settled on the panels. When *Ciona* spp. was sufficiently abundant, up to 30 juveniles were sampled on each panel and preserved in 100% ethanol. To optimize DNA extraction success, the largest juvenile in the cell was sampled, and successive adjacent cells were sampled until the maximum number of 30 cells was reached. DNA extraction was performed with the NucleoSpin^®^ 96 Tissue Kit according to the manufacturer's protocol (Macherey‐Nagel, Germany) with an additional treatment for removing RNA (10 μl of a stock solution of 25 mg/ml of RNase was added for 20 min after the tissue lysis step).

For juveniles sampled in 2013, we used the degenerate primers developed by Nydam and Harrison (2007) that have been shown to be reliable for distinguishing the two species in the study populations (Bouchemousse, Lévêque, et al., 2016). Species identification is based on a PCR‐RFLP protocol (i.e., enzymatic digestion of the PCR product) detailed in Nydam and Harrison (2011). Because amplification success was low for juveniles sampled in 2014, we developed species‐specific primers that increased the barcoding success significantly. These species‐specific primer pairs each amplify mtDNA of only one of the two species. The PCR fragment includes the region with the restriction site targeted with the degenerate nonspecific primers previously used. Thus, both PCR‐RFLP and direct PCR essays can be carried out with these primers. The forward and reverse primer sequences for amplifying *C. robusta* are, respectively, CiA‐mtCOIshort‐F: 5′‐ACAGTTTATCCTCCTTTATCTGCA‐3′ and CiA‐mtCOIshort‐R: 5′‐TGGATCTCTTCTCCCATTCGG‐3′. The forward and reverse primer sequences specific to *C. intestinalis* are, respectively, CiB‐mtCOIshort‐F: 5′‐CTTTGCATTTAGCTGGGGTTTC‐3′, CiB‐mtCOIshort‐R: 5′‐AGGATCCCTTCTTCTGTTAGGA‐3′. The amplifications with these primers were carried out in a total reaction volume of 20 μl, with 5 μl of template DNA diluted to 1:10, 1× buffer (Thermoprime, ABGene^®^), 0.2 mmol/L of each dNTP, 1.5 mmol/L of MgCl_2_, 0.05 μg/μl of bovine serum albumin, 0.05 μmol/L of each primer, and 0.2 U of Taq Polymerase (Thermoprime, ABGene^®^).

A touchdown PCR program was used, starting with 5 min at 95°C followed by 5 cycles with 30 s at 95°C, 30 s at an initial temperature of 56°C (then decreasing by 1°C per cycle), 30 s at 72°C and then 30 cycles at 95°C for 30 s, 52°C for 30 s, 72°C for 30 s, and a final elongation step at 72°C for 10 min. Species determination was easily carried out on an agarose gel (2%) because CiA‐mtCOIshort primers amplify a fragment of 306 base pairs (bp) in *C. robusta* and CiB‐mtCOIshort primers amplify a fragment of 239 bp in *C. intestinalis*. Both nonamplification and the PCR product size can be used to determine the species. Note that every individual was amplified using the two PCR primer pairs to check for consistency of the results.

### 2.3 Statistical analyses

We analyzed the number of juveniles settled per dm^2^ (i.e., juvenile density). We first investigated the annual settlement dynamics of *Ciona* spp. in each marina (i.e., from March of year *y* to March of year *y* + 1). Assuming that each major settlement event follows a Gaussian distribution, the number and characteristics (e.g., time of settlement) of discrete settlement events of juveniles were estimated using a modal decomposition analysis carried out with the R package MIXDIST (Green & Macdonald, 1985; R Development Core Team, 2014). This package contains maximum‐likelihood‐based methods to find the best fit between the observed data and a mixture of Gaussian distributions (Green & Macdonald, 1985). The density data were smoothed using a weighted moving average at the third order to rule out irregularities (Frontier & Pichod‐Viale, 1991). Prior to the modal decomposition analysis, the normality of each dataset modified by the weighted moving average was tested using a Shapiro test in R (the *shapiro.test* function). A significant deviation from normality is a prerequisite before attempting to find the best mixture of Gaussian curves explaining the data.

To compare the settlement rate among marinas and settlement periods, we used a two‐way analysis of variance (ANOVA) on the average density value computed across the month showing the highest density of juveniles (*m*) and the preceding and following months (*m* − 1 and *m *+* *1). For months characterized by an overlap between two settlements events within a calendar year, density values were divided by two. “Marinas” (*n* = 4) and “settlement periods” (*n* = 4) were considered as random factors. Prior to the ANOVA, the values of density were transformed using the square root function to meet the normality and homoskedasticity conditions (i.e., using the Shapiro and Levene tests, respectively). For factors showing a significant effect, pairwise comparisons were carried out with Student–Newman–Keuls (SNK) post hoc tests.

Following the DNA‐based species identification, except in Roscoff, where only *C. intestinalis* was found during the 2013–2014 survey, the same analyses were carried out for each species separately (i.e., modal decomposition, comparison of the intensity of settlement among sites, and settlement periods). The density of each of study species was computed for each panel and each month by multiplying the density value of *Ciona* spp. by the relative proportion of *C. robusta* (or *C. intestinalis*) obtained with the DNA‐based species identification.

## 3 Results

### 3.1 Settlement analyses of *Ciona* spp. reveal two settlement periods within a year

**Figure 2:**
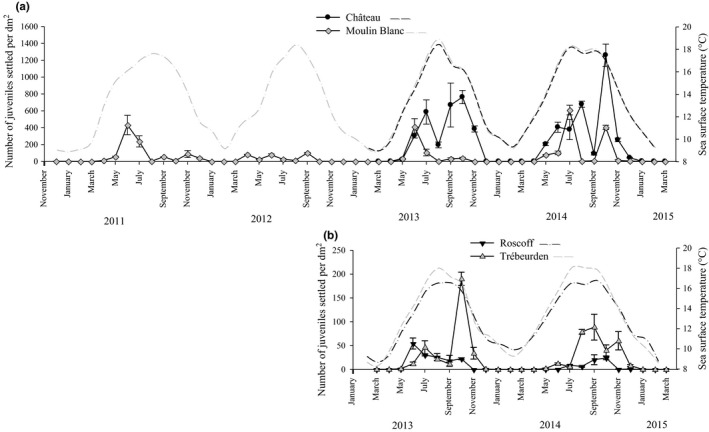
Monthly variation (mean ± standard error, *n* = 3) in the density of juveniles of *Ciona* spp. recorded on settlement panels (solid line) and of the daily average sea surface temperature (dotted line) recorded using a temperature data logger (TidbiT^®^v2) in the four study marinas (a) in the Bay of Brest (Château and Moulin Blanc marinas) and (b) along the northern coast of Brittany (Roscoff and Trébeurden marinas). Note the difference in scale for the *Y*‐axis between (a) and (b)

The settlement surveys carried out over 52 months in the Moulin Blanc marina, and over 25 months in the three other marinas, showed important changes in the number of juveniles observed monthly over the study period (Figure 2; Tables S2 and S3 for details). In all marinas, juveniles were generally observed from May to June until November–December. Practically, no juveniles were observed from January to March. In addition, regardless of the year or the marina, the distribution of the density of *Ciona* spp. juveniles differed significantly from a normal distribution (Fig. S1). For each study year, modal decomposition analyses clearly identified two Gaussian curves corresponding to two major periods of settlement with a short overlap. The average time of each settlement period (i.e., determined by the mode of the Gaussian curve) is provided in Table 1. Observed distribution and Gaussian curves for *Ciona* spp. are shown in Fig. S1, with parameters characterizing the Gaussian curves detailed in Table S4. Over a given study year, the first settlement period covers juveniles settled between April and August (spring–summer) and the second settlement period corresponds to juveniles settled between August and December (late summer–autumn; Figure 2, Table 1).

**Table 1:** Mean settlement period for each settlement event identified over the course of the study (i.e., 52 months) for *Ciona* spp. and for *Ciona robusta* and *Ciona intestinalis* separately

Marina	Settlement period label	Mean settlement period (month)		
		*Ciona* spp.	*C. robusta*	*C. intestinalis*
Moulin Blanc	1st‐2011	5.8 June‐11		
	2nd‐2011	10.4 October‐11		
	1st‐2012	16.8 May‐12		
	2nd‐2012	20.4 August‐12		
	1st‐2013	29.6 June‐13	29.8 June‐13	29.6 June‐13
	2nd‐2013	33.1 September‐13	32.3 August‐13	33.1 September‐13
	1st‐2014	42.3 June‐14	42.2 June‐14	42.3 June‐14
	2nd‐2014	45.6 October‐14	45.5 September‐14	45.6 October‐14
Château	1st‐2013	30.4 June‐13	31.0 July‐13	30.3 June‐13
	2nd‐2013	33.3 September‐13	33.7 October‐13	33.3 September‐13
	1st‐2014	42.4 June‐14	42.4 June‐14	42.5 June‐14
	2nd‐2014	45.5 October‐14	45.6 October‐14	45.6 October‐14
Roscoff	1st‐2013	29.8 June‐13	ND	29.8 June‐13
	2nd‐2013	32.4 August‐13	ND	32.4 August‐13
	1st‐2014	42.8 July‐14	ND	42.8 July‐14
	2nd‐2014	45.0 September‐14	ND	45.0 September‐14
Trébeurden	1st‐2013	30.6 July‐13	30.8 July‐13	30.4 June‐13
	2nd‐2013	33.6 October‐13	33.5 September‐13	33.6 October‐13
	1st‐2014	42.8 July‐14	44.7 September‐14	41.3 May‐14
	2nd‐2014	44.4 August‐14	46.7 Nov‐14	44.1 August‐14

The unit for the settlement period is the month, with values ranging thus from 0 (December 2010) to 51 (March 2015). Settlement periods were identified using modal decomposition analyses carried out separately for each year on the monthly distribution of the mean juvenile density on panels (*n* = 3). The settlement period indicated is the modal value of each identified Gaussian curve. Details of the results of the modal decomposition analyses are given in Figure S1 and Table S4 for *Ciona* spp. and in Figure S2 and Table S5 for the two species separately. Note that no *Ciona robusta* juveniles were identified in the Roscoff marina.

### 3.2 Variations in *Ciona* spp. settlement patterns among sites

Density data in Figure 2 show that settlement density varied among sites, with much higher values in the Château and Moulin Blanc marinas than in the Roscoff and Trébeurden marinas. For instance, in Château, the highest mean density values observed during the four settlement periods observed across the survey were 586.5 (±248.5 *SD*), 763.2 (±136.1 *SD*), 677.8 (±62.3 *SD*), and 1,259.4 (±229.2 *SD*) juveniles/dm^2^ in July 2013, October 2013, August 2014, and October 2014, respectively (Table S2). In comparison, in Roscoff, the density values were always lower, with a maximum mean density value of 54.4 (±20.1 *SD*) juv/dm^2^ for June 2013.

From March 2013 to March 2014, settlement density was not significantly different between settlement periods within sites (Table 2a) with the exception of the Moulin Blanc marina. There was however a highly significant difference between sites (*p *<* *.001; Table 2a). Pairwise comparisons (i.e., SNK post hoc tests) indicated that most of the differences were explained by higher values in the Château marina compared with the other marinas (Table 2a, Figure 2), particularly Trébeurden and Roscoff.

**Table 2:** Result of the two‐way ANOVA testing the effect of the settlement period (*n* = 4, 1st‐13: first settlement period in 2013, etc., see Table 1) and marina (*n* = 4 or 3, MBl: Moulin Blanc, Cha: Château, Ros: Roscoff, Treb: Trébeurden) on the density values corresponding to the month showing the highest density of juveniles and the preceding and following month (see Section 2), for (a) *Ciona* spp., (b) *Ciona robusta*, and (c) *Ciona intestinalis*

(a) Density of *Ciona* spp.					(b) Density of *C. robusta*				
Source of variance	*df*	SS	*F*	*p*‐Value	Source of variance	*df*	SS	*F*	*p*‐Value
Settlement period	3	6.1	0.02	.995	Settlement period	3	198.6	6.77	**.003**
Marina	3	5,094.9	18.02	**<.001**	Marina	2	249.0	12.74	**<.001**
Interaction	9	848.1	2.76	**.015**	Interaction	6	327.4	5.58	**<.001**
Residuals	128	2,363.3			Residuals	96	238.3		
**SNK interpretations**					**SNK interpretations**				
Within settlement period	1st‐13	Ros = Treb < MBl = Cha			Within settlement period	1st‐13	MBl = Cha = Treb		
	2nd‐13	Ros = Treb = MBl < Cha				2nd‐13	MBl < Cha = Treb		
	1st‐14	Ros = Treb < MBl = Cha				1st‐14	MBl = Cha = Treb		
	2nd‐14	Ros = Treb = MBl < Cha				2nd‐14	MBl = Treb < Cha		
Within marina	MBl	2nd‐13 < 1st‐13 = 1st‐14 = 2nd‐14			Within marina	MBl	All equal		
	Cha	All equal				Cha	1st‐13 = 2nd‐13 = 1st‐14 < 2nd‐14		
	Ros	All equal				Tre	1st‐13 < 2nd‐13 = 1st‐14 = 2nd‐14		
	Tre	All equal							

(c) Density of *C. intestinalis*				
Source of variance	*df*	SS	*F*	*p*‐Value
Settlement period	3	7.8	0.08	.969
Marina	3	4,533.7	48.84	**<.001**
Interaction	9	857.0	3.08	**.005**
Residuals	128	1,960.4		
**SNK interpretations**				
Within settlement period	1st‐13	Ros = Treb < MBl = Cha		
	2nd‐13	Ros = Treb = MBl < Cha		
	1st‐14	Ros = Treb < MBl = Cha		
	2nd‐14	Ros = Treb < MBl < Cha		
Within marina	MBl	2nd‐13 < 1st‐13 = 1st‐14 = 2nd‐14		
	Cha	All equal		
	Ros	All equal		
	Tre	All equal		

Degrees of freedom (*df*), sums of squares (SS), values of the statistic (*F*), and probability value (*p*‐value) are given. Significant *p*‐values are shown in bold. For significant factors, pairwise comparisons are provided (i.e., SNK post hoc tests).

The variations among settlement periods for a given site appeared to be moderate except for Moulin Blanc: There was a significant settlement period effect (1) for the 2013–2014 survey (Table 2a), attributed to the lower density values during the second settlement period in 2013 (i.e., always below 39.4 (±15.4 *SD*) juv/dm^2^ [mean density value for June 2013]) compared with the other surveys (Figure 2, Table S3); (2) and over a longer time series encompassing the density values from 2011 to 2014 (one‐way ANOVA: *F *=* *4.17, *df* = 7, *p *<* *.001). This result can be attributed to the particularly low settlement rates during spring 2012 compared with other periods (SNK post hoc interpretations: 2nd‐11 = 1st‐12 = 2nd‐12 = 2nd‐13 < 1st‐11 = 1st‐13 = 1st‐14 = 2nd‐14). After the low densities observed in spring 2011 and 2012 (Table S2), the intensity of spring settlement steadily increased in 2013 and 2014.

### 3.3 Comparison of settlement patterns between *C. robusta* and *C. intestinalis*

**Figure 3:**
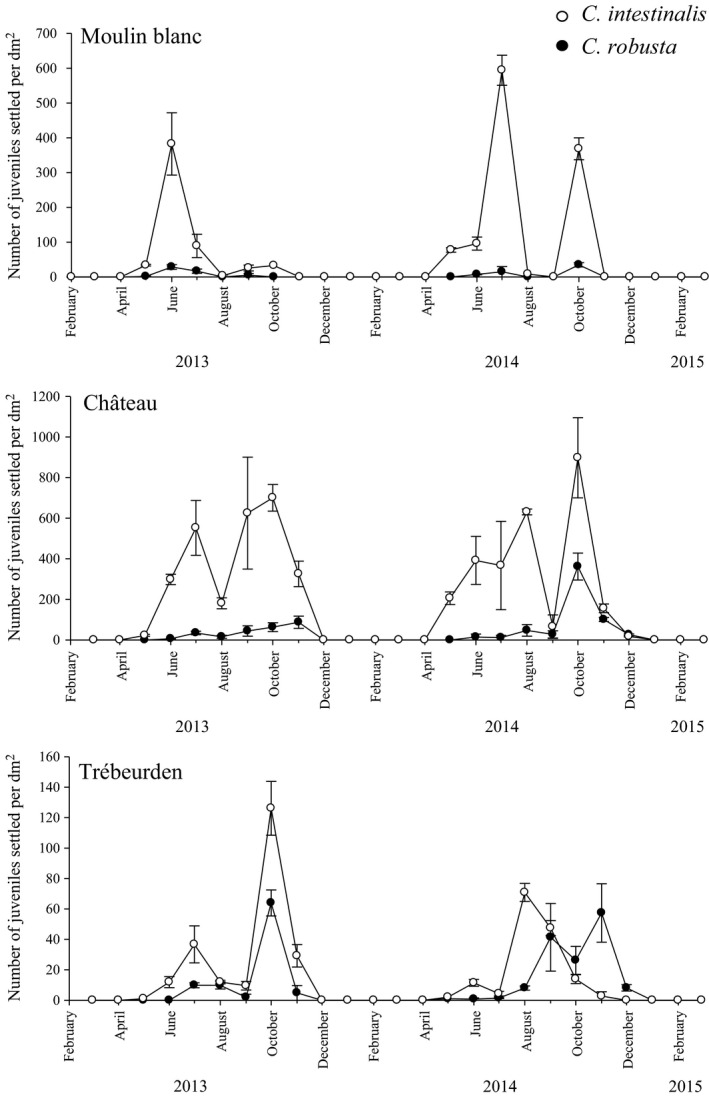
Monthly variation (mean ± standard error, *n* = 3) in the density of juveniles of *Ciona robusta* (black circles) and *Ciona intestinalis* (white circles) in the three marinas where the two species were found living in syntopy. The three graphs have different scales for the *Y*‐axis

A total of 1,650 juveniles were successfully barcoded in 2013 and 2014 with ca. 17% of them identified as *C. robusta* according to the mtCOI diagnostic marker. Details of the number of juveniles successfully barcoded and the relative abundance of *C. robusta* per month and per marina are given in Table S3. Throughout the survey, juveniles of *C. robusta* were recorded in three of the four marinas surveyed, namely Trébeurden, Château, and Moulin Blanc, but surprisingly absent in Roscoff (over 272 juveniles barcoded). Adults of *C. robusta* were reported in 2012 in the Roscoff marina before starting the settlement panel experiment (Bouchemousse, Lévêque, et al., 2016). Figure 3 displays the juvenile density of the two species in the three marinas where they are in syntopy.

**Figure 4:**
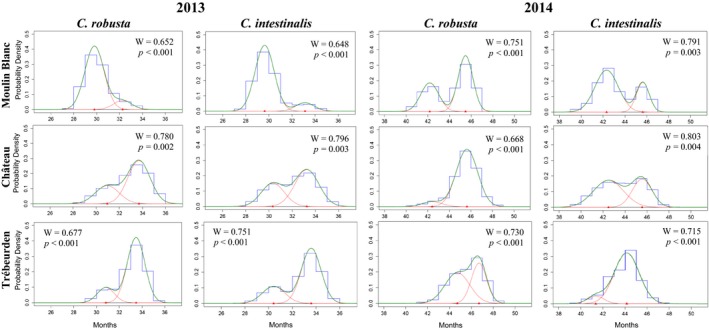
Graphical outcomes of the modal decomposition analysis for determining the number and properties of each major settlement events of *Ciona robusta* and *Ciona intestinalis* in 2013 and 2014 in the three marinas where the two species were found living in syntopy (i.e., Moulin Blanc, Château, and Trébeurden). The blue line (histogram) corresponds to the observed dataset (i.e., distribution of the density of juveniles for each month). The values were averaged per panels (*n* = 3 replicates) and smoothed using a running average over three classes (i.e., 3 months). Result of Shapiro–Wilk tests (*W*: value of the statistic, *p,* probability) on the deviation of the observed data from a normal distribution are provided: All distributions are significantly different from a normal distribution. Red and green curves show the outcome of the modal decomposition analysis and correspond to the Gaussian curves and the final adjusted curve, respectively. Characteristics of each Gaussian curve are detailed in Table S5

Modal decomposition analyses carried out for each species separately showed the same results as those for *Ciona* spp. Two settlement periods were observed in 2013 and 2014, for both study species: mainly during spring and early summer for the first settlement period and during end of summer and autumn for the second one (Figure 4). The average settlement time per settlement period and marina is provided in Table 1 for *C. intestinalis* and *C. robusta*. Details on the parameters of the modal decomposition outcomes are given in Table S5. Interestingly, the settlement timing of the two species was very similar regardless of the settlement period (Table 1, Figure 4). There was however one exception in Trébeurden in 2014: *C. robusta* settlement was delayed for at least 4 months, with mode values of the first and second settlement periods of 2014 being in September and November 2014, respectively, for *C. robusta,* but in May and August 2014, respectively, for *C. intestinalis* (Figure 4).

Throughout the 2013–2014 survey and regardless of the marina, the density of juveniles of *C. intestinalis* was much higher than those recorded for *C. robusta* (Figure 3). For example, in 2014 in Château (i.e., the marina showing the highest density values of *C. robusta,* Table S3), the average density value during the two settlement periods ranged from 11.5 (±19.9 *SD*) in July to 361.7 (±114.9 *SD*) juv/dm^2^ in October for *C. robusta*, but from 17.3 (±2.8 *SD*) in December to 897.7 (±197.5 *SD*) juv/dm^2^ in October for *C. intestinalis*. Result of the ANOVA on the settlement rate (i.e., density values of the month showing the highest density of juveniles and the preceding and following month) over 2013 and 2014 for *C. intestinalis* was comparable to those obtained for *Ciona* spp. (Table 2b, Figure 3): a strong and significant site effect explained by the extremely high density values in Château compared with the other marinas and an absence of significant effect of settlement period (except for Moulin Blanc). For *C. robusta*, different results were obtained: A significant settlement period effect was observed in addition to a significant site effect (Table 2c, Figure 3). These results are due to the higher densities of *C. robusta* juveniles (1) in Château, in particular, during the second settlement period of 2014 (average density value of 361.7 juv/dm^2^ (±114.9 *SD*) in October 2014) and (2) in Trébeurden where the settlement density was significantly higher during the last three settlement periods compared with the first one.

### 3.4 Comparison of density patterns at adult stages between *C. robusta* and *C. intestinalis*

**Figure 5:**
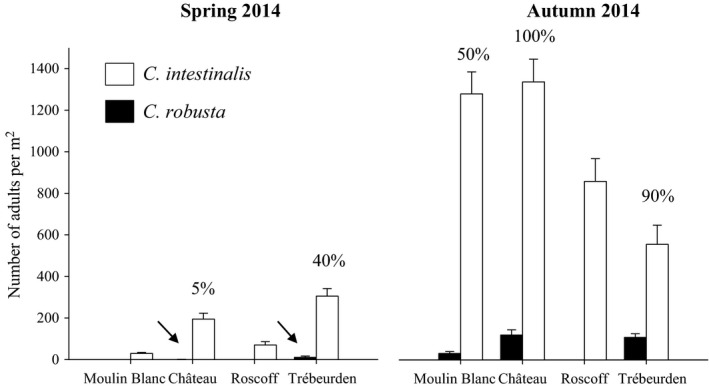
Density (mean ± standard error, *n* = 20) of *Ciona robusta* (black bars) and *Ciona intestinalis* (white bars) at the adult stage recorded in spring and autumn 2014 in the four marinas studied. Arrows indicate the presence of *C. robusta* (the relatively low values are difficult to discern on the large *Y*‐axis). The percentage of quadrats in which the two species were identified is indicated above the bar plots for each marina

Throughout the study, the same trend observed for juveniles was observed between adults of the two species sampled in 2014 in the three marinas where both species were present (Figure 5), that is, (1) a higher density for both species in autumn compared to spring generation, (2) a higher density for *C. intestinalis* compared to *C. robusta* whatever the season, and (3) an higher increase of *C. robusta* abundance within a year than that of *C. intestinalis*. For instance, in Château in autumn 2014, the average density of adults was 11 times higher (1,338 ind/m^2^ ±491 *SD*) for *C. intestinalis* compared with *C. robusta* (120 ind/m^2^ ±112 *SD*) and the increase in *C. robusta* density within a year was up to 100 times in autumn 2014 compared with spring 2014 while only seven times for *C. intestinalis*.

## 4 Discussion

### 4.1 Similar timing of settlement for the non‐native and native congeners in syntopic localities

In all marinas, except Roscoff, both *C. robusta* and *C. intestinalis* recruited on the settlement panels that we set out. When both species were present in a given marina and month, the juveniles of both species were found on each panel without any evidence of spatial clustering (i.e., relative proportions of the two species were similar on all three panels). That the two species are well mixed on experimental substrates reflects observations of adults growing under pontoons (see figure 1 in Bouchemousse, Lévêque, et al., 2016) and the density survey of adults in 2014. For instance, in autumn 2014, both species were identified in all of the quadrats sampled in Château (Figure 5). In the study area, the two species live in very tight syntopy at the juvenile and adult stages.

Based on modal decomposition analyses, in the three marinas where juveniles of both species settled, we identified two similar settlement periods for each species in each study year*:* One settlement period was observed at the end of spring–early summer and a second one in late summer–early fall. This result corroborates observations of simultaneous gamete production by the adults during spring and autumn for both *Ciona* species in syntopic localities in the English Channel (Bouchemousse, Lévêque, et al., 2016).

Previous studies of these two *Ciona* species have reported juvenile settlement dynamics for one or the other of the two species independently. Although these studies use the names “*Ciona intestinalis*” or “*C. intestinalis* type B” and “*C. intestinalis* type A” (Nydam & Harrison, 2007), we were able to determine the identity of the species examined according to the geographical location of the study (see Supplementary Note in Bouchemousse, Bishop, et al., 2016 for a detailed map). For *C. intestinalis*, our findings are in agreement with results obtained in Nova Scotia and Prince Edward Island (Carver, Chisholm, & Mallet, 2003; Howes, Herbinger, Darnell, & Vercaemer, 2007; Vercaemer, Sephton, Nicolas, Howes, & Keays, 2011), in Sweden (Dybern, 1965), and in a Danish fjord (Petersen & Svane, 1995), where two settlement periods were identified and are similar to those found in our study. In the English Channel, information is available on adults only (i.e., in Plymouth, Orton, 1914, 1920), but also fit with the assumption of two generations, and thus two settlement periods, per year. For *C. robusta*, our study is the first to document settlement patterns in the NE Atlantic. There are however data available for San Francisco Bay, another region where the species has been presumably introduced: Two settlement periods are also observed, one occurring in August and one in October–November (Blum et al., 2007). Other data documenting the life cycle of *C. robusta* are based on temporal surveys of adults in the Mediterranean Sea: Caputi et al. (2015) suggested the existence of more than two generations with reproductive adults present throughout the year, including during winter months. Similarly, three or four generations have been documented for different regions of the Mediterranean Sea (i.e., Adriatic Sea, eastern and western Mediterranean Sea, see Dybern, 1965 and references therein). Altogether, our results and data from the literature suggest that, regardless of the geographical region, at least two generations occur over a calendar year for both *Ciona* species, with variation in the settlement period for *C. robusta* probably explained by different environmental conditions (e.g., temperature).

Our data clearly indicate a similar number of generations per year and settlement periods in the English Channel for the two congeneric species, indicating no major shift in the phenology of the two species. Consequently, competitive interactions for space may occur during juvenile growth in localities where the recruitment is intense for both species, such as the Château marina (Figure 3). Such early competition may influence adult abundance because recruitment success is a key feature in space monopolization for sessile organisms (e.g., between ascidian species; Rius, Potter, Aguirre, & Stachowicz, 2014). There is however one exception to the general pattern observed, in Trébeurden in 2014 (Table 1, Figure 3), where recruitment of *C. robusta* juveniles was delayed by at least 4 months compared with their native congeners. We have no clear explanation for this singularity, especially considering that we did not observe any obvious differences in gamete production in either species in this marina (Bouchemousse, Lévêque, et al., 2016). However, this finding may indicate the onset of a phenological shift between the two species in this marina, which is also one of the few marinas where the NNS *C. robusta* can have higher relative abundance than *C. intestinalis* at the adult stage (Bouchemousse, Lévêque, et al., 2016). In any case, we conclude that phenological shifts are not driving any potential competitive exclusion between the NNS *C. robusta* and its native congener in situations of syntopy.

### 4.2 Can larval supply or environmental conditions explain variation in settlement success between marinas?

The overall settlement timing was similar for the two species, but we observed important differences in settlement rate of juveniles at different levels, in particular between marinas. For *C. intestinalis*, a much higher number of juveniles were observed in the marinas of the Bay of Brest (i.e., Château and Moulin Blanc) than in the two marinas located on the northern coast of Brittany (i.e., Roscoff and Trébeurden). This trend for a site effect was not observed for *C. robusta,* despite the higher numbers of juveniles in the Château marina compared with the other marinas. Similar observations were also found at the adult stage during surveys carried out in spring and autumn 2014 (Figure 5): Higher densities for *C. intestinalis* were observed in the two marinas in the Bay of Brest, and for *C. robusta* in Château.

Local hydrodynamic features can have a strong impact on the density and the aggregation of *Ciona* spp. juveniles (Havenhand & Svane, 1991), and more generally on marine invertebrates with a bentho‐pelagic life cycle (Hunt & Scheibling, 1997). Both the Château and Moulin Blanc marinas are located in the Bay of Brest, a semiclosed system, which may contribute to retaining larvae close to the sites where they have been released and thus enhance larval supply near resident adult populations (Gaines & Bertness, 1992). Moreover, close to estuaries of important rivers (i.e., Aulne and Elorn, Figure 1a), food supply is also likely to be higher in the marinas of the Bay of Brest compared with Trébeurden and Roscoff. Conversely, the Roscoff marina is an open marina located in the Bay of Morlaix in which tidal currents are important, thereby potentially preventing local larval retention and settlement events. Significant larval exports out of this bay have been demonstrated using an analytical hydrodynamic model for the mollusk *C. fornicata,* another NNS in the Bay of Morlaix (Rigal et al., 2010). Larval export was suggested as a possible explanation for the low proliferation of this NNS in the bay. Similar processes may occur for *Ciona* species, although their larval stage is short and their ability to settle quickly (within 12–24 hr under laboratory conditions) following fertilization. Fine‐scale hydrodynamic models covering the study marinas and ports are unfortunately not available. Dedicated models need to be developed to examine in further detail the role of larval supply/larval export to/from urban marine environments.

In contrast to *C. intestinalis*, we found a significant settlement period effect on the density of juveniles for *C. robusta* (Table 2b). As pictured in Figure 3, the abundance of settled *C. robusta* juveniles increased over time within a year in two of the three study marinas where this species was present (i.e., Trébeurden and Château). This increase in *C. robusta* abundance within a year was also documented for adults (Figure 5) with up to a 10‐fold increase in the density of *C. robusta* in autumn 2014 compared with spring 2014. This increase is most likely due to warmer conditions from July to September (Figure 2), that are more favorable to *C. robusta* recruitment, considered to be a warm‐temperate species compared with the cold‐temperate species *C. intestinalis* (Bouchemousse, Lévêque, et al., 2016; Procaccini, Affinito, Toscano, & Sordino, 2011). This presumed difference in temperature preferences may also explain the disappearance of *C. robusta* in the Roscoff marina after 2012 (i.e., 1 year after its building). Adult individuals of *C. robusta* have indeed been observed in 2012 in this marina (Table S1) but have completely disappeared in 2013 and 2014 (Table S1, Figure 5). Note that more recent surveys (July 2016) confirmed the absence of *C. robusta* in this locality (L. Leveque & F. Viard, unpublished data). This marina is located in the coldest part of the Brittany coastline (Gallon et al., 2014), and its maximal temperatures are below 17°C (Figure 2). These temperature conditions may favor reproduction and sustainable population establishment of *C. intestinalis* at the expense of *C. robusta* (Bouchemousse, Lévêque, et al., 2016).

Other abiotic factors such as large changes in surface salinity due to rainfall and river inputs are known to influence the dynamics of ascidian communities established under pontoons and floating docks (Lambert & Lambert, 1998, 2003). In our study, we observed dramatic changes in the density of *Ciona* spp. juveniles in Moulin Blanc over the 4 years survey (Figure 2): The settlement rate in 2012 was particularly low compared with other years. This is most likely due to the almost complete collapse of adult *Ciona* populations in this marina (L.L., personal diving observations) following the exceptional rainfalls that occurred in April (i.e., 211.4 mm during April 2012, compared to average of 91.9 ± 35.2 mm (April 1981–April 2010, source: http://www.meteo-bretagne.fr/climatologie-normales-Brest-Guipavas). *Ciona* populations have progressively recovered since then, despite other episodes of high rainfall and collapse of adult populations in winters 2013 and 2014 (Bouchemousse, Lévêque, et al., 2016). Similar population collapses notably due to rainfall events have been recently documented in another introduced ascidian, *S. plicata* (Pineda, Turon, Perez‐Portela, & Lopez‐Legentil, 2016 and references therein). Although located close by (5 km), the Château marina seems less exposed to such events, perhaps because it is further away from the estuaries of two important rivers (i.e., Aulne and Elorn, Figure 1a) than Moulin Blanc. Similarly, Trébeurden and Roscoff being located in fully marine parts of the Brittany coastline are even less affected by rainfall. Apart from stochastic events, whose effects may have an impact over several years, juvenile populations of *Ciona* spp. in Moulin Blanc seem to be comparable to Château in terms of density as shown by the recruitment peak in spring 2013 and 2014.

Altogether, the site and settlement effects documented here for juveniles are similar to those observed for adults, indicating a positive feedback loop between the density of adults and of juveniles in *Ciona* spp.—for the two species together and independently. The changes over time (i.e., between seasons) are however more pronounced in *C. robusta*, suggesting that warmer conditions may facilitate the settlement of the NNS and subsequently increase its recruitment success compared with its native congener.

### 4.3 Does competition with its native congener limit the invasiveness of *C. robusta*?

Despite its advantage during the warmer season, we documented a consistently low recruitment success in *C. robusta* than in *C. intestinalis*. For example, the maximum value observed over the whole dataset was in Château in October 2014, with a density value of 361.6 juv/dm^2^, a value more than two times lower than for *C. intestinalis* (898.7 juv/dm^2^) at the same place and date. The same result was observed for the adult stage in all sampling periods (Figure 5).

Inhibition of settlement and subsequent growth by a superior competitor can strongly influence the abundance of species, as shown in numerous cases for marine invertebrates (Davis, Butler, & Vanaltena, 1991; Grosberg, 1981), including in ascidians (Rius et al., 2009; Svane & Young, 1989). For example, using ex situ experiments, Rius et al. (2009) showed that the presence of *S. plicata* juveniles, a non‐native ascidian in Australia, increased juvenile mortality and reduced the growth of the native ascidian *Microcomus squamiger*, probably via competition for food. The opposite situation, that is, negative influence of native species on the settlement of NNS, has also been reported. For example, in Spain (Mediterranean Sea), predation of larvae by juveniles of the native mussel *M. galloprovincialis* was shown to stimulate settlement avoidance close to mussels of two non‐native ascidians *S. plicata* and *Microcomus squamiger* (Ordonez et al., 2013). Under the competition hypothesis, marinas with the highest density of one *Ciona* species should have the lowest density of the other species. However, the exact opposite was observed in our study, that is, the Château marina showed the highest density values for juveniles and adults of both species. This suggests that competitive interactions are not major drivers of the population dynamics in these two species (i.e., food or space are not limiting) and that environmental and/or biotic factors in this site favor the population expansion of both species. Moreover, similar values of the density of juveniles have been observed for *C. robusta* in another region, the San Francisco Bay, where it has been introduced but does not coexist with *C. intestinalis*, with a maximum density of ca. 300 juveniles/dm^2^ (Blum et al., 2007). Our density data for *C. intestinalis* are also similar to those documented for *C. intestinalis* in Nova Scotia (e.g., up to 1,450/dm^2^; Vercaemer et al., 2011) and in Prince Edward Island (e.g., up to 4,800 individuals/dm^2^; Ramsay, Davidson, Bourque, & Stryhn, 2009), two localities in which *C. intestinalis* is considered as a NNS.

Considering that competition between the two species at the settlement stage does not currently appear to be a significant driving factor of population abundance, and because *C. robusta* settlement and adult density both increase in autumn, the abundance of this species is expected to increase from year to year. However, the exact opposite trend is observed, with a particularly low adult density and juvenile settlement rate of this species in spring. For instance, in Trébeurden, the highest density value for *C. robusta* during the second settlement event of 2013 was twice as low as that of *C. intestinalis*, but the adult density value was 27 times lower for *C. robusta* compared with *C. intestinalis* in spring 2014. We suggest that the autumn settlement of *C. robusta* is successful, but subsequent growth and/or survival is limited. The NNS seems to suffer from unfavorable environmental conditions during the winter season (i.e., coldest temperatures and strong variation of salinity, as explained above for *Ciona* spp.), which may either reduce juvenile growth and/or cause adult mortality before the onset of their reproductive period in spring. This finding is consistent with known differences in environmental preferences between the two species (i.e., *C. robusta* is considered to be a warm‐temperate species and *C. intestinalis* is a more cold‐temperate species, as mentioned before). Thus, the winter season seems to have a lesser impact on the mortality of *C. intestinalis* populations than on those of *C. robusta* and may therefore explain the much lower adult density and thus settlement density of *C. robusta* than *C. intestinalis* in the spring.

Altogether, our results suggest that competitive exclusion at early stages between the NNS *C. robusta* and its native congener in situation of syntopy is unlikely to play a major role in determining their patterns of coexistence in the wild. Regarding the density of adults recorded in 2014, variations in settlement rate between marinas and settlement period indicate a positive feedback loop between adult and juveniles for both *Ciona* species. The observed annual density variations, more pronounced for *C. robusta*, suggest that environmental variations in abiotic parameters (i.e., temperature and salinity) play a critical role in the population dynamics of the NNS: Warmer seasons seem to facilitate the settlement of *C. robusta,* and conversely, winter seasons limit its expansion by inducing higher mortality rate than the native species. However, biological invasions are dynamic processes in which “boom and bust” dynamics are often documented, and which require long‐term surveys to be properly examined (Strayer, Eviner, Jeschke, & Pace, 2006). Considering the documented warming of seawater in the English Channel (Gallon et al., 2014), mortality and/or reduced growth of *C. robusta* may be lowered and its competitive ability over *C. intestinalis* may increase. Long‐term field monitoring associated with experimental studies investigating the influence of environmental conditions on the survival and growth of both *Ciona* species are needed to determine the fate of the NNS *C. robusta* in the English Channel.

## Conflict of Interest

None declared.
