# Supplementary material for: Do settlement dynamics influence competitive interactions between an alien tunicate and its native congener?
Source: Ecol Evol. 2016 Dec 17;7(1):200–13. doi: 10.1002/ece3.2655 (PMC5213624; doi:10.1002/ece3.2655)
Supplement: Supplementary file 1 [file ECE3-7-200-s001.docx]

**Supporting Information**

**Do settlement dynamics influence competitive interactions between an alien tunicate and its native congener?**

Sarah Bouchemousse^1,2^, Laurent Lévêque^1,3^ and Frédérique Viard^1,2,*^

Table S1. **Proportions of the non-native species *Ciona robusta* relative to all *Ciona* spp. individuals recorded in spring and autumn in 2012, 2013 and 2014** with the average value over the whole study period (data from Bouchemousse et al. 2016). Bouchemousse et al. (2016) showed that the relative proportion varies across seasons, with high values observed locally in autumn only (e.g. Trébeurden, Château).

| Marina | Spring 2012 | Autumn 2012 | Spring 2013 | Autumn 2013 | Spring 2014 | Autumn 2014 | Mean - Spring | Mean - Autumn | Overall Mean |
| --- | --- | --- | --- | --- | --- | --- | --- | --- | --- |
| Moulin Blanc | 18.5 | 42.7 | 35.8 | 2.2 | 0.3 | 0 | 18.2 | 15.0 | 16.6 |
| Château | 3.4 | 19.1 | 0 | 6 | 0 | 3.3 | 1.1 | 9.5 | 5.3 |
| Roscoff | 8.8 | 2.5 | 0 | 0 | 0 | 0 | 2.9 | 0.8 | 1.9 |
| Trébeurden | 6.6 | 61.1 | 6.7 | 43.2 | 6.4 | 16.4 | 6.6 | 40.2 | 23.4 |

Table S2. **Dataset obtained in 2011, 2012 and early 2013 in the Moulin Blanc marina**

For each series of panels retrieved in Moulin Blanc between December 2010 (month 0) and February 2013 (month 26), the table gives the density (number per dm^2^) of juveniles per panel (P1, P2, P3).

|  |  |  | Density (number per dm^2^)  of juveniles per panel | | |
| --- | --- | --- | --- | --- | --- |
| Marina | Month |  | P1 | P2 | P3 |
| Moulin Blanc | 0-Dec-10 |  | 2.0 | 1.0 | 3.1 |
|  | 1-Jan-11 |  | 3.1 | 0.0 | 0.0 |
|  | 2-Feb-11 |  | 0.0 | 0.0 | 0.0 |
|  | 3-Mar-11 |  | 0.0 | 0.0 | 0.0 |
|  | 4-Apr-11 |  | 10.2 | 12.2 | 12.2 |
|  | 5-May-11 |  | 38.8 | 59.2 | 67.3 |
|  | 6-Jun-11 |  | 303.0 | 333.6 | 659.1 |
|  | 7-Jul-11 |  | 110.2 | 272.4 | 331.6 |
|  | 8-Aug-11 |  | 2.0 | 1.0 | 4.1 |
|  | 9-Sep-11 |  | 55.1 | 57.1 | 57.1 |
|  | 10-Oct-11 |  | 3.1 | 7.1 | 17.3 |
|  | 11-Nov-11 |  | 147.9 | 10.2 | 107.1 |
|  | 12-Dec-11 |  | 53.1 | 41.8 | 28.6 |
|  | 13-Jan-12 |  | 0.0 | 0.0 | 0.0 |
|  | 14-Feb-12 |  | 0.0 | 0.0 | 1.0 |
|  | 15-Mar-12 |  | 1.0 | 2.0 | 0.0 |
|  | 16-Apr-12 |  | 88.8 | 83.7 | 73.5 |
|  | 17-May-12 |  | 12.2 | 35.7 | 25.5 |
|  | 18-Jun-12 |  | 54.1 | 88.8 | 92.8 |
|  | 19-Jul-12 |  | 9.2 | 23.5 | 45.9 |
|  | 20-Aug-12 |  | 13.3 | 10.2 | 15.3 |
|  | 21-Sep-12 |  | 97.9 | 115.3 | 89.8 |
|  | 22-Oct-12 |  | 2.0 | 3.1 | 2.0 |
|  | 23-Nov-12 |  | 0.0 | 0.0 | 2.0 |
|  | 24-Dec-12 |  | 0.0 | 0.0 | 1.0 |
|  | 25-Jan-13 |  | 1.0 | 0.0 | 0.0 |
|  | 26-Feb-13 |  | 0.0 | 0.0 | 0.0 |

Table S3. **Detailed dataset obtained in 2013 and 2014 in the four study marinas**

For each marina and for each series of panels retrieved between March 2013 (month 27) and March 2015 (month 51), the table gives the density (number per dm^2^) of juveniles per panel (P1, P2, P3), the number of juveniles used for DNA-based species identification (Nind) and the associated genotyping success, the proportion of *C. robusta* juveniles relative to all *Ciona* spp. juveniles per panels (note that for a few months, the sampled juveniles collected were pooled over panels and only the value for the three panels combined is provided (in italics)). The density of *C. intestinalis* and *C. robusta* juveniles per panel was computed from the juvenile density cumulated over the two species and the relative proportion of the two species at the juvenile stage.

|  |  |  | Density (number per dm^2^) of juveniles per panels | | |  | Number of juveniles genotyped | |  | Relative proportion of *C. robusta* juveniles over all *Ciona* spp. juveniles | | | |  | Density (number of juveniles per dm^2^) of *C. robusta* | | |
| --- | --- | --- | --- | --- | --- | --- | --- | --- | --- | --- | --- | --- | --- | --- | --- | --- | --- |
| Marina | Month |  | P1 | P2 | P3 |  | Nind | % of success |  | P1 | P2 | P3 | Total |  | P1 | P2 | P3 |
| Moulin | 27-Mar-13 |  | 1.0 | 0.0 | 0.0 |  |  |  |  |  |  |  |  |  |  |  |  |
| Blanc | 28-Apr-13 |  | 3.1 | 3.1 | 0.0 |  | 3 | 0.0% |  |  |  |  |  |  |  |  |  |
|  | 29-May-13 |  | 28.6 | 40.8 | 35.7 |  | 30 | 96.7% |  | 0% | 10% | 0% | 3% |  | 0.0 | 4.1 | 0.0 |
|  | 30-Jun-13 |  | 591.8 | 264.3 | 376.5 |  | 51 | 84.3% |  |  |  |  | *7%* |  | *41.3* | *18.4* | *26.3* |
|  | 31-Jul-13 |  | 119.6 | 166.5 | 31.0 |  | 69 | 92.8% |  |  |  |  | *16%* |  | *18.7* | *26.0* | *4.8* |
|  | 32-Aug-13 |  | 8.2 | 1.0 | 1.0 |  | 10 | 100.0% |  | 0% | 0% | 0% | 0% |  | 0.0 | 0.0 | 0.0 |
|  | 33-Sep-13 |  | 57.1 | 18.4 | 17.3 |  | 40 | 97.5% |  | 25% | 8% | 0% | 10% |  | 14.3 | 1.5 | 0.0 |
|  | 34-Oct-13 |  | 28.6 | 32.6 | 57.1 |  | 12 | 58.3% |  |  | 0% |  | 0% |  |  | 0.0 |  |
|  | 35-Nov-13 |  | 0.0 | 0.0 | 0.0 |  |  |  |  |  |  |  |  |  |  |  |  |
|  | 36-Dec-13 |  | 0.0 | 0.0 | 1.0 |  |  |  |  |  |  |  |  |  |  |  |  |
|  | 37-Jan-14 |  | 0.0 | 0.0 | 0.0 |  |  |  |  |  |  |  |  |  |  |  |  |
|  | 38-Feb-14 |  | 0.0 | 0.0 | 0.0 |  |  |  |  |  |  |  |  |  |  |  |  |
|  | 39-Mar-14 |  | 0.0 | 0.0 | 0.0 |  |  |  |  |  |  |  |  |  |  |  |  |
|  | 40-Apr-14 |  | 4.1 | 2.0 | 2.0 |  | 4 | 0.0% |  |  |  |  |  |  |  |  |  |
|  | 41-May-14 |  | 74.5 | 89.9 | 68.4 |  | 45 | 51.1% |  | 0% | 0% | 0% | 0% |  | 0.0 | 0.0 | 0.0 |
|  | 42-Jun-14 |  | 125.1 | 120.4 | 63.3 |  | 47 | 91.5% |  | 6% | 7% | 8% | 7% |  | 7.8 | 8.6 | 4.9 |
|  | 43-Jul-14 |  | 586.7 | 717.3 | 523.4 |  | 47 | 100.0% |  | 0% | 6% | 0% | 2% |  | 0.0 | 44.8 | 0.0 |
|  | 44-Aug-14 |  | 2.0 | 8.2 | 0.0 |  | 8 | 75.0% |  |  | 0% |  | 0% |  |  | 0.0 |  |
|  | 45-Sep-14 |  | 6.1 | 14.3 | 5.1 |  |  |  |  |  |  |  |  |  |  |  |  |
|  | 46-Oct-14 |  | 414.2 | 354.0 | 440.8 |  | 45 | 100.0% |  | 7% | 13% | 7% | 9% |  | 27.6 | 47.2 | 29.4 |
|  | 47-Nov-14 |  | 3.1 | 31.6 | 1.8 |  | 12 | 0.0% |  |  |  |  |  |  |  |  |  |
|  | 48-Dec-14 |  | 8.2 | 1.0 | 7.1 |  |  |  |  |  |  |  |  |  |  |  |  |
|  | 49-Jan-15 |  | 0.0 | 0.0 | 3.1 |  |  |  |  |  |  |  |  |  |  |  |  |
|  | 50-Feb-15 |  | 0.0 | 0.0 | 0.0 |  |  |  |  |  |  |  |  |  |  |  |  |
|  | 51-Mar-15 |  | 0.0 | 0.0 | 0.0 |  |  |  |  |  |  |  |  |  |  |  |  |
| Château | 27-Mar-13 |  | 0.0 | 0.0 | 0.0 |  |  |  |  |  |  |  |  |  |  |  |  |
|  | 28-Apr-13 |  | 2.0 | 0.0 | 1.0 |  |  |  |  |  |  |  |  |  |  |  |  |
|  | 29-May-13 |  | 31.6 | 20.4 | 15.3 |  | 20 | 95.0% |  | 0% | 0% | 0% | 0% |  | 0.0 | 0.0 | 0.0 |
|  | 30-Jun-13 |  | 296.9 | 263.2 | 352.0 |  | 57 | 89.5% |  |  |  |  | *2%* |  | *5.8* | *5.2* | *6.9* |
|  | 31-Jul-13 |  | 313.2 | 798.9 | 647.4 |  | 57 | 89.5% |  |  |  |  | *6%* |  | *18.4* | *47.0* | *38.1* |
|  | 32-Aug-13 |  | 230.6 | 126.5 | 230.6 |  | 30 | 96.7% |  | 10% | 0% | 10% | 7% |  | 23.1 | 0.0 | 23.1 |
|  | 33-Sep-13 |  | 1142.7 | 618.6 | 244.9 |  | 57 | 100.0% |  | 0% | 14% | 18% | 9% |  | 0.0 | 88.4 | 43.2 |
|  | 34-Oct-13 |  | 844.8 | 606.1 | 838.7 |  | 57 | 89.5% |  | 13% | 6% | 6% | 8% |  | 105.6 | 33.7 | 49.3 |
|  | 35-Nov-13 |  | 330.6 | 453.0 | 372.4 |  | 20 | 90.0% |  |  | 11% | 33% | 22% |  |  | 50.3 | 124.1 |
|  | 36-Dec-13 |  | 0.0 | 0.0 | 0.0 |  |  |  |  |  |  |  |  |  |  |  |  |
|  | 37-Jan-14 |  | 0.0 | 2.0 | 0.0 |  |  |  |  |  |  |  |  |  |  |  |  |
|  | 38-Feb-14 |  | 0.0 | 0.0 | 0.0 |  |  |  |  |  |  |  |  |  |  |  |  |
|  | 39-Mar-14 |  | 0.0 | 0.0 | 0.0 |  |  |  |  |  |  |  |  |  |  |  |  |
|  | 40-Apr-14 |  | 1.0 | 1.0 | 0.0 |  | 2 | 0.0% |  |  |  |  |  |  |  |  |  |
|  | 41-May-14 |  | 219.4 | 227.5 | 170.4 |  | 46 | 17.4% |  | 0% | 0% | 0% | 0% |  | 0.0 | 0.0 | 0.0 |
|  | 42-Jun-14 |  | 526.5 | 343.8 | 347.9 |  | 50 | 96.0% |  | 0% | 0% | 13% | 4% |  | 0.0 | 0.0 | 43.5 |
|  | 43-Jul-14 |  | 259.2 | 617.2 | 258.1 |  | 45 | 100.0% |  | 0% | 0% | 13% | 4% |  | 0.0 | 0.0 | 34.4 |
|  | 44-Aug-14 |  | 614.2 | 738.7 | 680.5 |  | 46 | 100.0% |  | 0% | 13% | 6% | 7% |  | 0.0 | 98.5 | 42.5 |
|  | 45-Sep-14 |  | 118.4 | 96.9 | 62.2 |  | 50 | 50.0% |  | 6% | 13% | 100% | 12% |  | 7.4 | 12.1 | 62.2 |
|  | 46-Oct-14 |  | 1010.1 | 1461.1 | 1307.0 |  | 45 | 100.0% |  | 33% | 33% | 20% | 29% |  | 336.7 | 487.0 | 261.4 |
|  | 47-Nov-14 |  | 233.6 | 234.7 | 300.0 |  | 46 | 100.0% |  | 40% | 38% | 40% | 39% |  | 93.5 | 88.0 | 120.0 |
|  | 48-Dec-14 |  | 35.7 | 44.9 | 50.0 |  | 45 | 84.4% |  | 58% | 55% | 67% | 61% |  | 20.8 | 24.5 | 33.3 |
|  | 49-Jan-15 |  | 3.1 | 4.1 | 2.0 |  |  |  |  |  |  |  |  |  |  |  |  |
|  | 50-Feb-15 |  | 1.0 | 0.0 | 0.0 |  |  |  |  |  |  |  |  |  |  |  |  |
|  | 51-Mar-15 |  | 0.0 | 0.0 | 0.0 |  |  |  |  |  |  |  |  |  |  |  |  |
| Roscoff | 27-Mar-13 |  | 0.0 | 0.0 | 0.0 |  |  |  |  |  |  |  |  |  |  |  |  |
|  | 28-Apr-13 |  | 0.0 | 0.0 | 0.0 |  |  |  |  |  |  |  |  |  |  |  |  |
|  | 29-May-13 |  | 1.0 | 1.0 | 1.0 |  | 2 | 100.0% |  | 0% |  | 0% | 0% |  | 0.0 |  | 0.0 |
|  | 30-Jun-13 |  | 77.5 | 44.9 | 40.8 |  | 57 | 91.2% |  | 0% | 0% | 0% | 0% |  | 0.0 | 0.0 | 0.0 |
|  | 31-Jul-13 |  | 33.7 | 29.6 | 27.5 |  | 57 | 100.0% |  | 0% | 0% | 0% | 0% |  | 0.0 | 0.0 | 0.0 |
|  | 32-Aug-13 |  | 27.5 | 12.2 | 37.8 |  | 30 | 100.0% |  | 0% | 0% | 0% | 0% |  | 0.0 | 0.0 | 0.0 |
|  | 33-Sep-13 |  | 40.8 | 5.1 | 8.2 |  | 23 | 100.0% |  | 0% | 0% | 0% | 0% |  | 0.0 | 0.0 | 0.0 |
|  | 34-Oct-13 |  | 18.4 | 23.5 | 25.5 |  | 25 | 84.0% |  | 0% | 0% | 0% | 0% |  | 0.0 | 0.0 | 0.0 |
|  | 35-Nov-13 |  | 0.0 | 0.0 | 0.0 |  |  |  |  |  |  |  |  |  |  |  |  |
|  | 36-Dec-13 |  | 0.0 | 0.0 | 1.0 |  |  |  |  |  |  |  |  |  |  |  |  |
|  | 37-Jan-14 |  | 0.0 | 0.0 | 0.0 |  |  |  |  |  |  |  |  |  |  |  |  |
|  | 38-Feb-14 |  | 0.0 | 0.0 | 0.0 |  |  |  |  |  |  |  |  |  |  |  |  |
|  | 39-Mar-14 |  | 0.0 | 0.0 | 0.0 |  |  |  |  |  |  |  |  |  |  |  |  |
|  | 40-Apr-14 |  | 0.0 | 0.0 | 0.0 |  |  |  |  |  |  |  |  |  |  |  |  |
|  | 41-May-14 |  | 1.0 | 0.0 | 0.0 |  | 1 | 0.0% |  |  |  |  |  |  |  |  |  |
|  | 42-Jun-14 |  | 0.0 | 1.0 | 0.0 |  | 1 | 0.0% |  |  |  |  |  |  |  |  |  |
|  | 43-Jul-14 |  | 10.2 | 6.1 | 10.2 |  | 23 | 95.7% |  | 0% | 0% | 0% | 0% |  | 0.0 | 0.0 | 0.0 |
|  | 44-Aug-14 |  | 7.1 | 9.2 | 3.1 |  | 10 | 20.0% |  | 0% | 0% |  | 0% |  | 0.0 | 0.0 |  |
|  | 45-Sep-14 |  | 30.6 | 27.5 | 33.7 |  | 32 | 100.0% |  |  | 0% | 0% | 0% |  |  | 0.0 | 0.0 |
|  | 46-Oct-14 |  | 31.6 | 20.4 | 20.4 |  | 45 | 57.8% |  | 0% | 0% |  | 0% |  | 0.0 | 0.0 | 0.0 |
|  | 47-Nov-14 |  | 0.0 | 0.0 | 1.0 |  | 1 | 0.0% |  |  |  |  |  |  |  |  |  |
|  | 48-Dec-14 |  | 2.0 | 1.0 | 0.0 |  | 2 | 100.0% |  |  |  |  | 0% |  |  |  | 0.0 |
|  | 49-Jan-15 |  | 0.0 | 0.0 | 0.0 |  |  |  |  |  |  |  |  |  |  |  |  |
|  | 50-Feb-15 |  | 0.0 | 0.0 | 0.0 |  |  |  |  |  |  |  |  |  |  |  |  |
|  | 51-Mar-15 |  | 0.0 | 0.0 | 0.0 |  |  |  |  |  |  |  |  |  |  |  |  |
| Trébeurden | 27-Mar-13 |  | 1.0 | 0.0 | 0.0 |  |  |  |  |  |  |  |  |  |  |  |  |
|  | 28-Apr-13 |  | 0.0 | 1.0 | 0.0 |  |  |  |  |  |  |  |  |  |  |  |  |
|  | 29-May-13 |  | 3.1 | 1.0 | 1.0 |  | 2 | 50.0% |  |  | 0% |  | 0% |  |  | 0.0 |  |
|  | 30-Jun-13 |  | 5.1 | 17.3 | 13.3 |  | 20 | 80.0% |  | 0% | 0% | 0% | 0% |  | 0.0 | 0.0 | 0.0 |
|  | 31-Jul-13 |  | 26.5 | 40.8 | 72.4 |  | 63 | 84.1% |  | 25% | 27% | 17% | 23% |  | 6.6 | 10.9 | 12.1 |
|  | 32-Aug-13 |  | 18.4 | 20.4 | 26.5 |  | 49 | 95.9% |  | 29% | 53% | 50% | 45% |  | 5.2 | 10.9 | 13.3 |
|  | 33-Sep-13 |  | 3.9 | 12.7 | 18.0 |  | 19 | 78.9% |  | 0% | 0% | 33% | 20% |  | 0.0 | 0.0 | 6.0 |
|  | 34-Oct-13 |  | 217.3 | 181.6 | 171.4 |  | 79 | 97.5% |  | 30% | 27% | 46% | 34% |  | 64.4 | 48.9 | 78.6 |
|  | 35-Nov-13 |  | 24.5 | 19.4 | 58.2 |  | 18 | 100.0% |  | 0% | 0% | 25% | 17% |  | 0.0 | 0.0 | 14.5 |
|  | 36-Dec-13 |  | 2.0 | 0.0 | 1.0 |  |  |  |  |  |  |  |  |  |  |  |  |
|  | 37-Jan-14 |  | 0.0 | 0.0 | 0.0 |  |  |  |  |  |  |  |  |  |  |  |  |
|  | 38-Feb-14 |  | 0.0 | 0.0 | 0.0 |  |  |  |  |  |  |  |  |  |  |  |  |
|  | 39-Mar-14 |  | 0.0 | 0.0 | 0.0 |  |  |  |  |  |  |  |  |  |  |  |  |
|  | 40-Apr-14 |  | 0.0 | 0.0 | 0.0 |  |  |  |  |  |  |  |  |  |  |  |  |
|  | 41-May-14 |  | 3.1 | 3.1 | 0.0 |  | 6 | 50.0% |  |  | 33% |  | 33% |  |  | 1.0 |  |
|  | 42-Jun-14 |  | 12.2 | 9.6 | 14.9 |  | 23 | 39.1% |  | 0% | 25% | 0% | 11% |  | 0.0 | 2.4 | 0.0 |
|  | 43-Jul-14 |  | 4.1 | 4.1 | 9.2 |  | 21 | 95.2% |  | 20% | 40% | 20% | 25% |  | 0.8 | 1.6 | 1.8 |
|  | 44-Aug-14 |  | 69.4 | 86.7 | 80.6 |  | 48 | 100.0% |  | 13% | 6% | 13% | 10% |  | 8.7 | 5.4 | 10.1 |
|  | 45-Sep-14 |  | 58.2 | 142.8 | 65.3 |  | 45 | 97.8% |  | 29% | 60% | 33% | 41% |  | 16.6 | 85.7 | 21.8 |
|  | 46-Oct-14 |  | 41.8 | 59.2 | 19.4 |  | 44 | 88.6% |  | 73% | 67% | 44% | 64% |  | 30.7 | 39.5 | 8.6 |
|  | 47-Nov-14 |  | 25.5 | 91.8 | 63.3 |  | 41 | 100.0% |  | 100% | 100% | 87% | 95% |  | 25.5 | 91.8 | 54.8 |
|  | 48-Dec-14 |  | 11.2 | 9.2 | 4.1 |  | 17 | 100.0% |  | 100% | 100% | 100% | 100% |  | 11.2 | 9.2 | 4.1 |
|  | 49-Jan-15 |  | 1.0 | 0.0 | 0.0 |  |  |  |  |  |  |  |  |  |  |  |  |
|  | 50-Feb-15 |  | 0.0 | 0.0 | 0.0 |  |  |  |  |  |  |  |  |  |  |  |  |
|  | 51-Mar-15 |  | 0.0 | 0.0 | 0.0 |  |  |  |  |  |  |  |  |  |  |  |  |

Figure S1. **Decomposition of normal distributions for determining the number and properties of each major settlement event of *Ciona* spp. in 2013 and 2014 in the four study marinas, with two additional years (2011, 2012) for Moulin Blanc**.

The blue line (histogram) corresponds to the observed dataset (i.e. distribution of the density of juveniles for each month). The values were averaged over panels (n = 3 replicates) and smoothed using a running average over three classes (i.e. 3 months). Results of Shapiro-Wilk test (W: value of the statistic, *P*: probability) on the deviation of the observed data from a normal distribution are provided: all distributions are significantly different from a normal distribution. Red and green curves represent the outcome of the modal decomposition analysis, and correspond to the Gaussian curves identified and the final adjusted curve, respectively. Characteristics of each Gaussian curve are detailed in Table S4. Computations were carried out using the R package MIXDIST.

| **2011**  Probability Density  **Moulin Blanc** | **2012** | **2013** | **2014** |
| --- | --- | --- | --- |
| 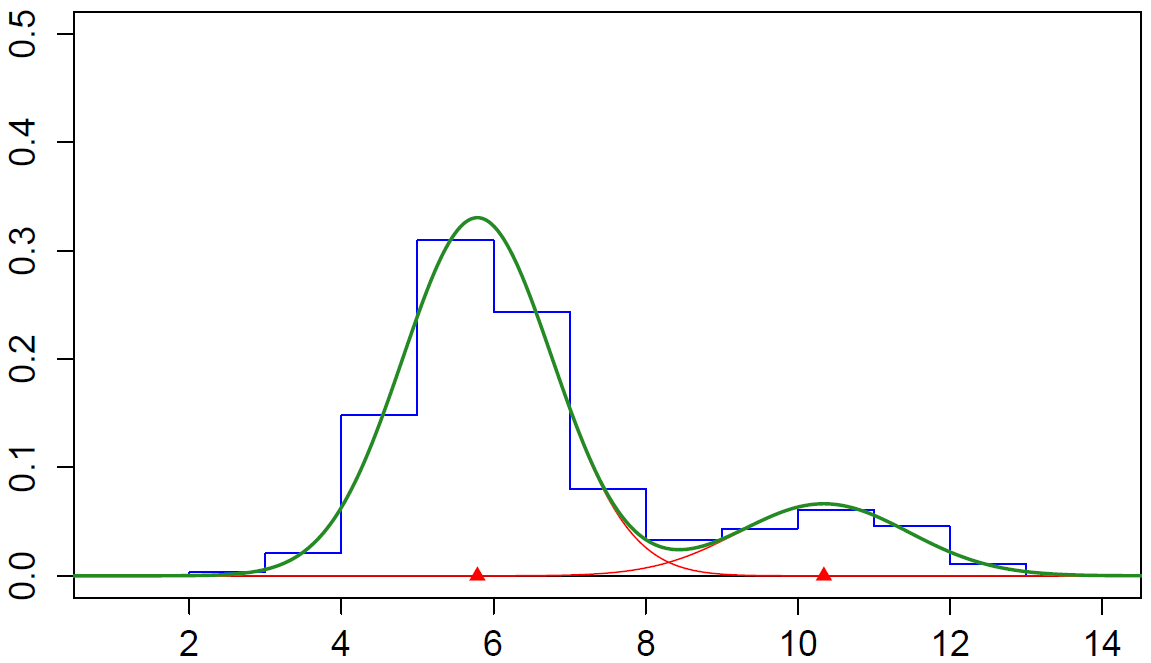  W = 0.750  *P* = 0.001  Month | 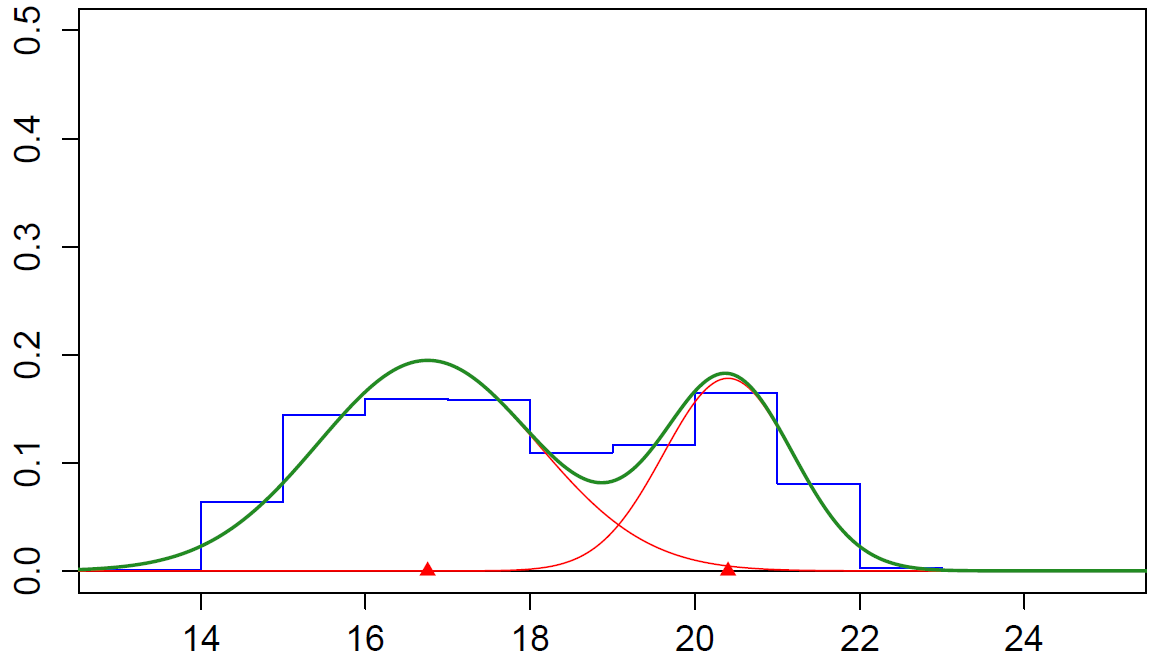  Month  Probability Density  W = 0.801  *P* = 0.004  **Château** | 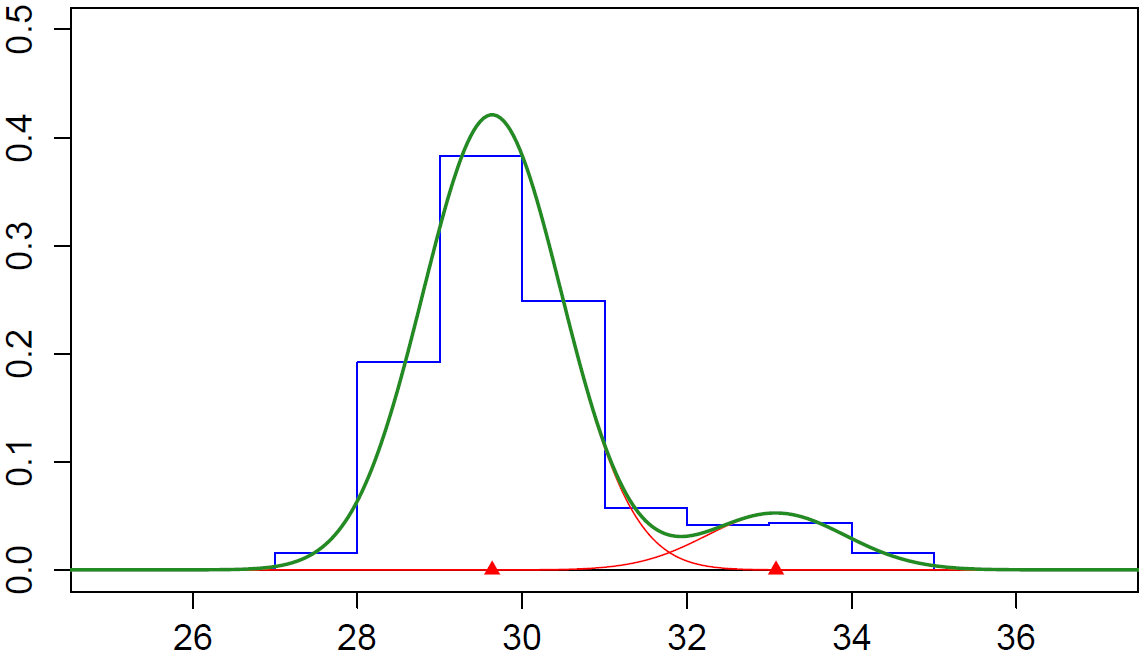  W = 0.695  *P* < 0.001 | 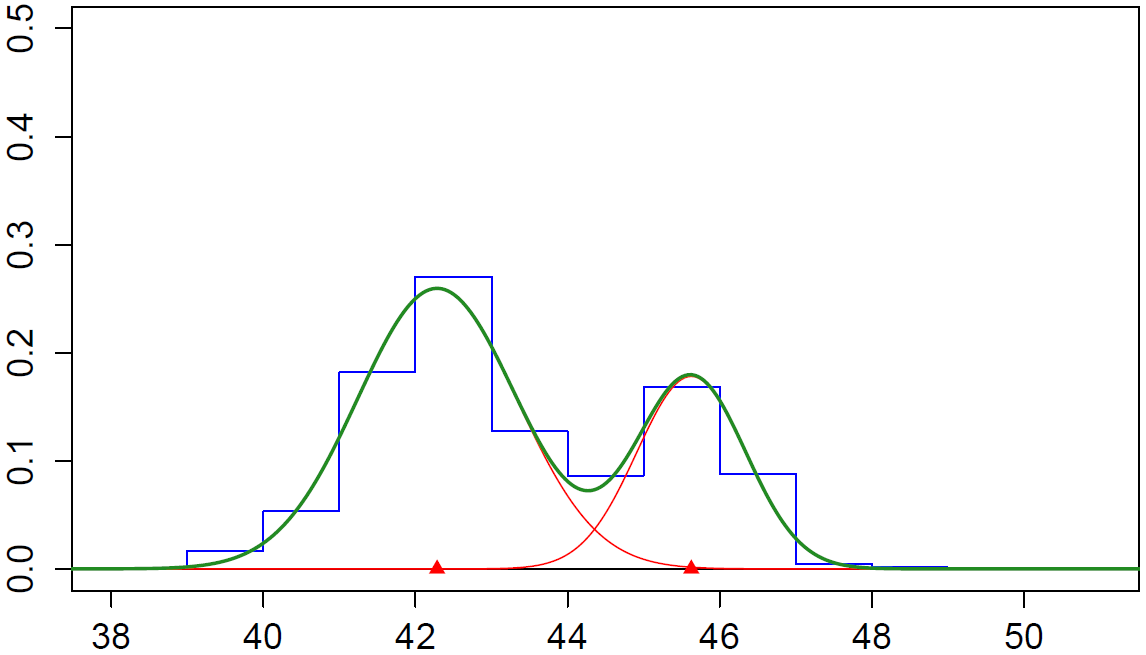  W = 0.802  *P* = 0.004 |
|  | Probability Density  **Roscoff** | 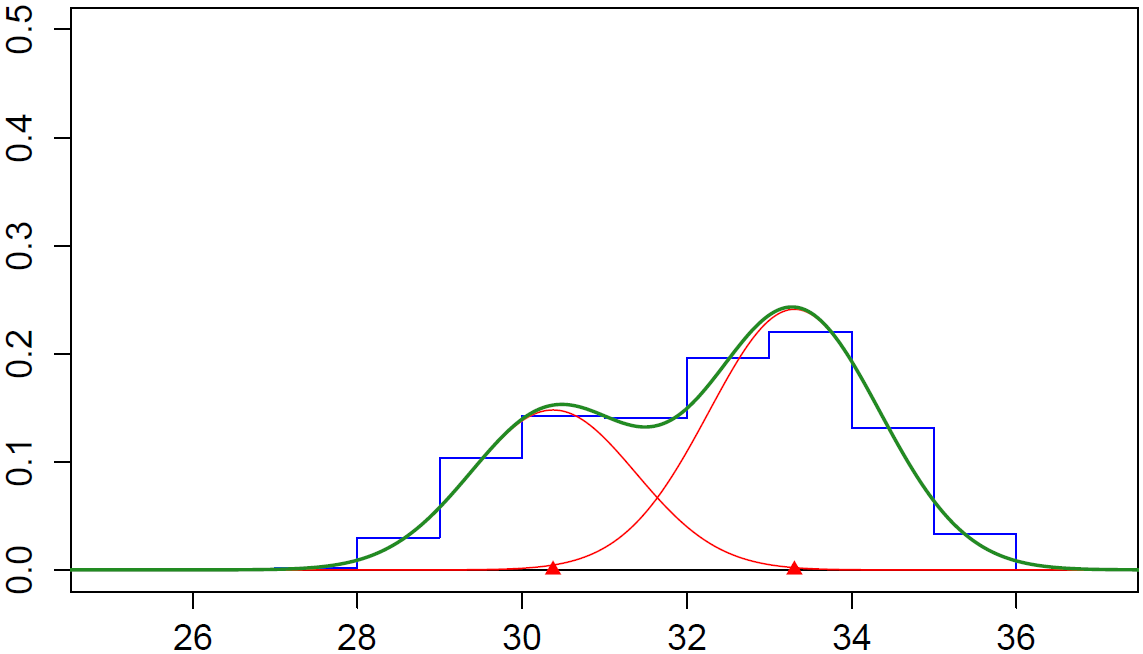  W = 0.820  *P* = 0.009 | 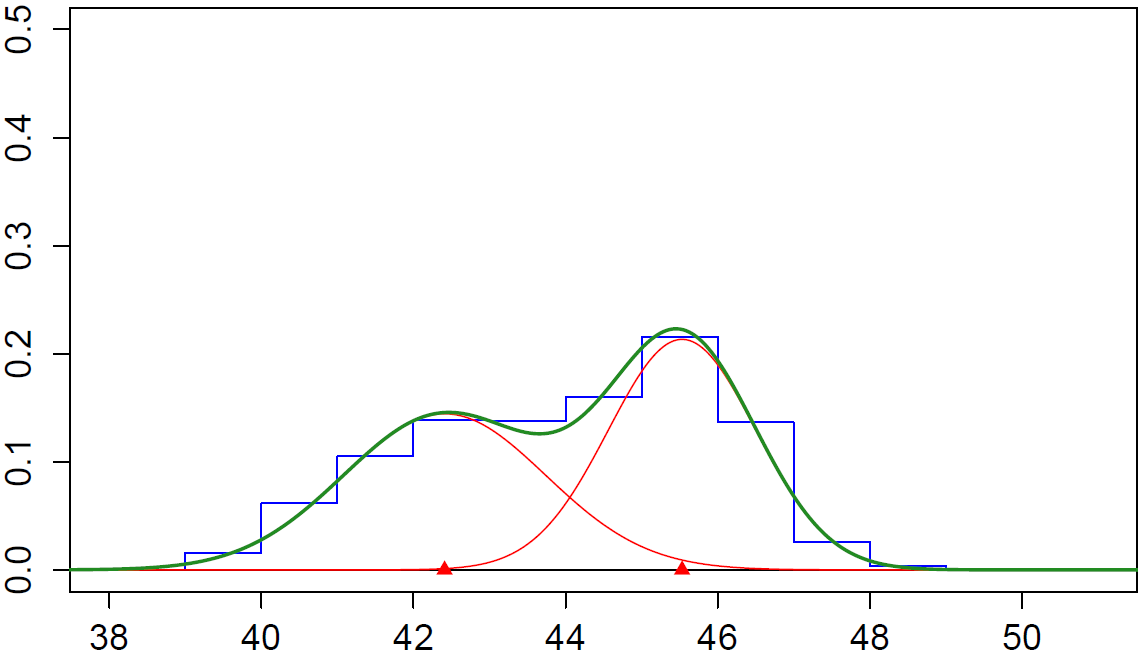  W = 0.827  *P* = 0.008 |
|  | Probability Density  **Trébeurden** | 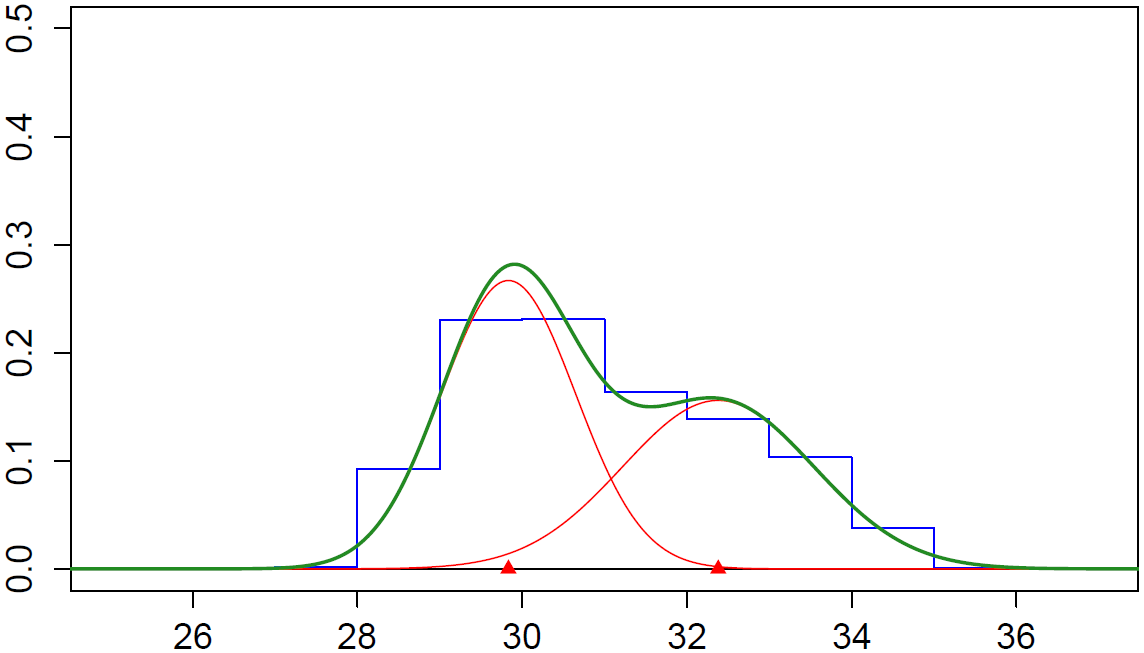  W = 0.814  *P* = 0.009 | 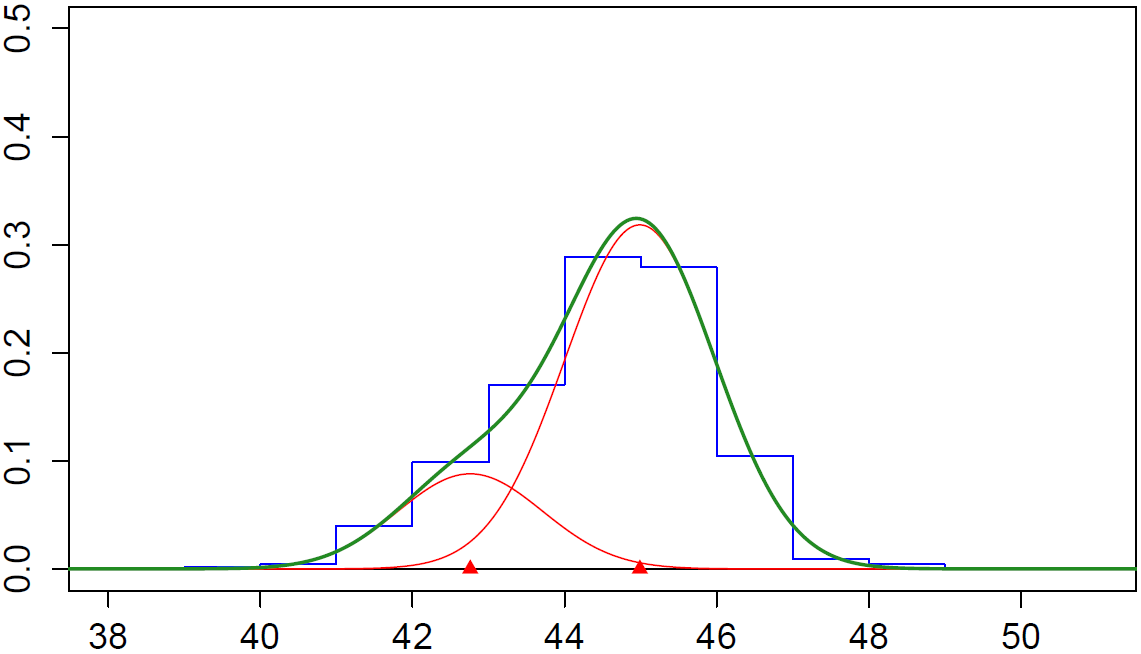  W = 0.724  *P* < 0.001 |
|  |  | 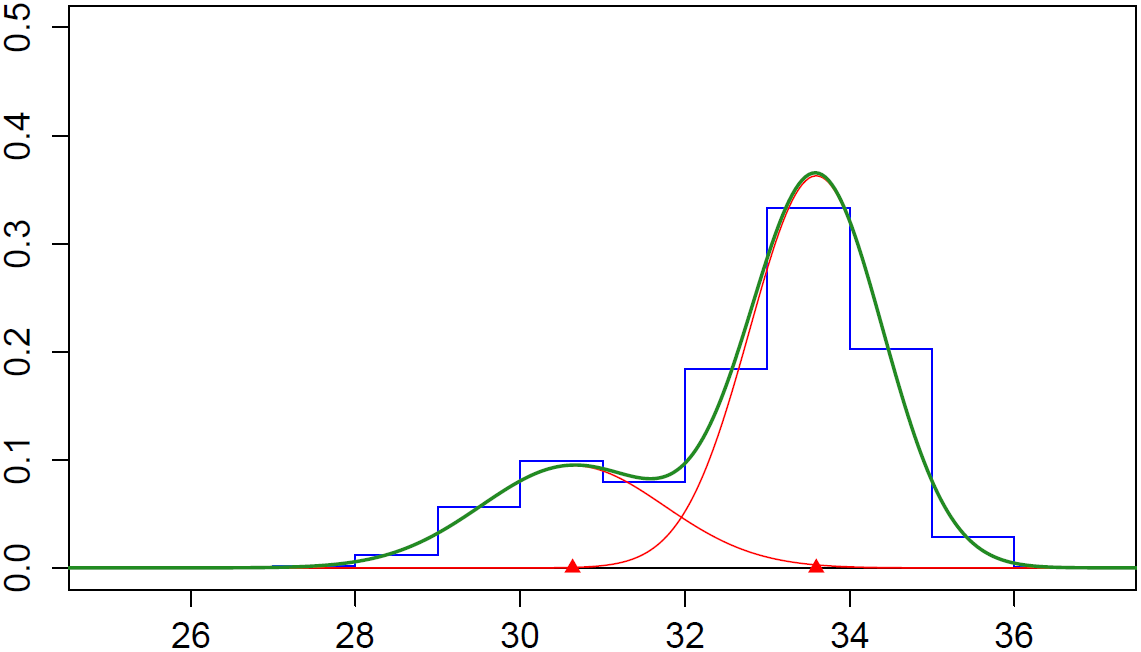  W = 0.755  *P* = 0.002 | 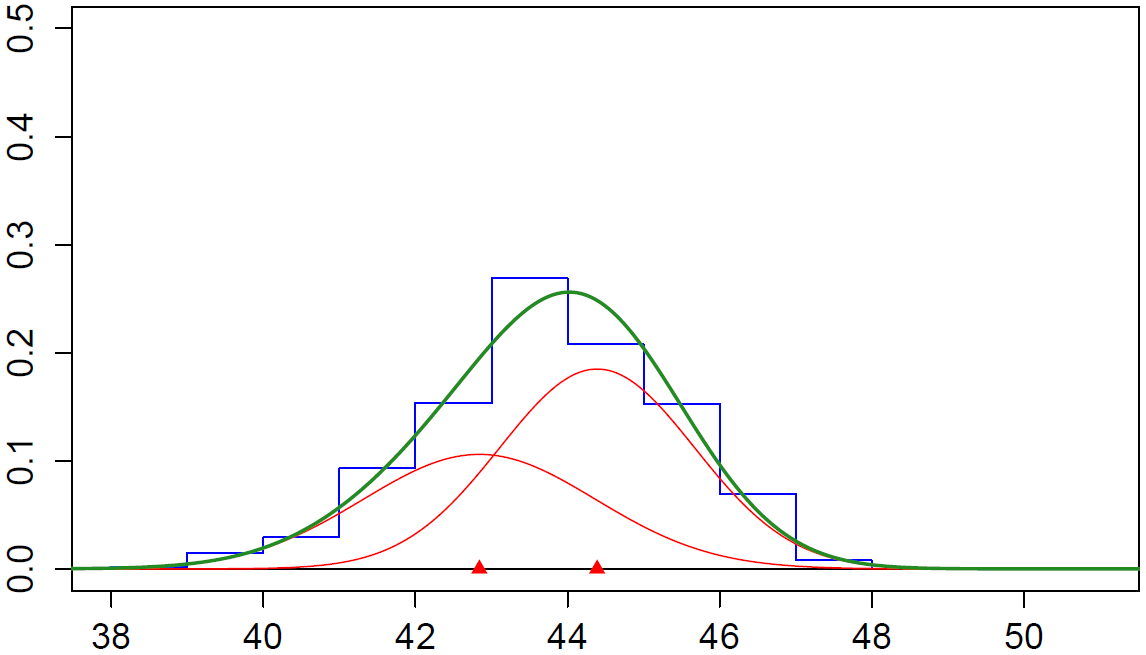  Month  W = 0.805  *P* = 0.006 |

Month

Table S4. **Parameters of the Gaussian curves, estimated with the R package MIXDIST, describing the** **annual settlement dynamics of *Ciona* spp. in the four study marinas.**

Modal decompositions were carried out for each year independently. Values for the proportion, mode and standard deviation (SD) are provided for each of the two Gaussian curves that gave the best fit with the observed data for each year (Fig. S1). The goodness of fit was tested with a χ^2^ test (df: degree of freedom, χ^2^: value of the statistic, *P*-value: probability associated with the χ^2^ test).

| **Year** | **Marina** | **Parameters** | **Modal decomposition** | | **χ^2^ statistic** |
| --- | --- | --- | --- | --- | --- |
|  |  |  | **1** | **2** |  |
| 2011 | Moulin | Proportion | 0.81 | 0.19 | df = 7 |
|  | Blanc | Mode | 5.79 | 10.35 | χ^2^ = 8.15 |
|  |  | SD | 0.98 | 1.11 | *P-*value =0.42 |
|  |  |  |  |  |  |
| 2012 | Moulin | Proportion | 0.65 | 0.35 | df = 5 |
|  | Blanc | Mode | 16.76 | 20.40 | χ^2^ = 8.43 |
|  |  | SD | 1.33 | 0.79 | *P-*value =0.30 |
|  |  |  |  |  |  |
| 2013 | Moulin | Proportion | 0.89 | 0.11 | df = 6 |
|  | Blanc | Mode | 29.64 | 33.08 | χ^2^ = 3.98 |
|  |  | SD | 0.84 | 0.84 | *P-*value =0.68 |
|  | Château | Proportion | 0.37 | 0.63 | df = 6 |
|  |  | Mode | 30.37 | 33.30 | χ^2^ = 10.09 |
|  |  | SD | 1.00 | 1.04 | *P-*value =0.10 |
|  | Roscoff | Proportion | 0.55 | 0.45 | df = 6 |
|  |  | Mode | 29.83 | 32.38 | χ^2^ = 2.53 |
|  |  | SD | 0.82 | 1.16 | *P-*value =0.87 |
|  | Trébeurden | Proportion | 0.26 | 0.74 | df = 6 |
|  |  | Mode | 30.64 | 33.59 | χ^2^ = 2.29 |
|  |  | SD | 1.11 | 0.81 | *P-*value =0. 90 |
|  |  |  |  |  |  |
| 2014 | Moulin | Proportion | 0.68 | 0.32 | df = 6 |
|  | Blanc | Mode | 42.28 | 45.63 | χ^2^ = 6.21 |
|  |  | SD | 1.05 | 0.72 | *P-*value =0.48 |
|  | Château | Proportion | 0.48 | 0.52 | df = 6 |
|  |  | Mode | 42.41 | 45.53 | χ^2^ = 9.68 |
|  |  | SD | 1.33 | 0.97 | *P-*value =0.16 |
|  | Roscoff | Proportion | 0.21 | 0.79 | df = 6 |
|  |  | Mode | 42.76 | 44.99 | χ^2^ = 0.72 |
|  |  | SD | 0.67 | 0.27 | *P-*value =0.99 |
|  | Trébeurden | Proportion | 0.41 | 0.59 | df = 7 |
|  |  | Mode | 42.84 | 44.39 | χ^2^ = 5.18 |
|  |  | SD | 1.53 | 1.28 | *P-*value =0.64 |

Table S5. **Parameters of Gaussian curves, estimated with the R package MIXDIST, describing the** **annual settlement dynamics of *Ciona robusta* (a) and *C. intestinalis* (b) in 2013 and 2014 in the three marinas where the two species were found living in syntopy (i.e. Moulin Blanc, Château and Trébeurden).** Graphical outcomes of this analysis are provided in Figure 4 of the main text. Modal decompositions were carried out for each year independently. Values for the proportion, mode and standard deviation (SD) are provided for each of the two Gaussian curves that gave the best fit with the observed data for each year (Fig. S1). The goodness of fit was tested with a χ^2^ test (df: degrees of freedom, χ^2^: value of the statistic, *P*-value: probability associated with the χ^2^ test).

(a)

| **Year** | **Marina** | **Parameters** | **Modal decomposition** | | **χ^2^ statistic** |
| --- | --- | --- | --- | --- | --- |
|  |  |  | **1** | **2** |  |
| 2013 | Moulin | Proportion | 0.88 | 0.12 | df = 4 |
|  | Blanc | Mode | 29.80 | 32.26 | χ^2^ = 0.62 |
|  |  | SD | 0.84 | 0.79 | *P-*value =0.96 |
|  | Château | Proportion | 0.31 | 0.69 | df = 6 |
|  |  | Mode | 30.96 | 33.70 | χ^2^ = 4.07 |
|  |  | SD | 1.03 | 0.96 | *P-*value =0.67 |
|  | Trébeurden | Proportion | 0.18 | 0.82 | df = 4 |
|  |  | Mode | 30.82 | 33.47 | χ^2^ = 0.91 |
|  |  | SD | 0.74 | 0.77 | *P-*value =0.92 |
|  |  |  |  |  |  |
| 2014 | Moulin | Proportion | 0.38 | 0.62 | df = 5 |
|  | Blanc | Mode | 42.17 | 45.48 | χ^2^ = 1.48 |
|  |  | SD | 0.82 | 0.68 | *P-*value =0.92 |
|  | Château | Proportion | 0.07 | 0.93 | df = 6 |
|  |  | Mode | 42.41 | 45.64 | χ^2^ = 2.68 |
|  |  | SD | 0.93 | 0.99 | *P-*value =0.85 |
|  | Trébeurden | Proportion | 0.55 | 0.45 | df = 5 |
|  |  | Mode | 44.73 | 46.70 | χ^2^ = 0.85 |
|  |  | SD | 1.13 | 0.70 | *P-*value =0.97 |

(b)

| **Year** | **Marina** | **Parameters** | **Modal decomposition** | | **χ^2^ statistic** |
| --- | --- | --- | --- | --- | --- |
|  |  |  | **1** | **2** |  |
| 2013 | Moulin | Proportion | 0.88 | 0.12 | df = 5 |
|  | Blanc | Mode | 29.62 | 33.12 | χ^2^ = 5.11 |
|  |  | SD | 0.82 | 0.81 | *P-*value =0.40 |
|  | Château | Proportion | 0.38 | 0.62 | df = 6 |
|  |  | Mode | 30.34 | 33.26 | χ^2^ = 7.69 |
|  |  | SD | 0.99 | 1.04 | *P-*value =0.21 |
|  | Trébeurden | Proportion | 0.26 | 0.74 | df = 6 |
|  |  | Mode | 30.42 | 33.59 | χ^2^ = 0.87 |
|  |  | SD | 0.96 | 0.83 | *P-*value =0.99 |
|  |  |  |  |  |  |
| 2014 | Moulin | Proportion | 0.72 | 0.28 | df = 6 |
|  | Blanc | Mode | 42.32 | 45.63 | χ^2^ = 6.36 |
|  |  | SD | 1.06 | 0.58 | *P-*value =0.49 |
|  | Château | Proportion | 0.59 | 0.41 | df = 7 |
|  |  | Mode | 42.44 | 45.51 | χ^2^ = 8.55 |
|  |  | SD | 1.37 | 0.89 | *P-*value =0.27 |
|  | Trébeurden | Proportion | 0.09 | 0.91 | df = 7 |
|  |  | Mode | 41.33 | 44.13 | χ^2^ = 4.72 |
|  |  | SD | 0.74 | 1.13 | *P-*value =0.68 |
